# Isolation and characterization of an atypical *LEA* gene (*IpLEA*) from *Ipomoea pes-caprae* conferring salt/drought and oxidative stress tolerance

**DOI:** 10.1038/s41598-019-50813-w

**Published:** 2019-10-16

**Authors:** Jiexuan Zheng, Huaxiang Su, Ruoyi Lin, Hui Zhang, Kuaifei Xia, Shuguang Jian, Mei Zhang

**Affiliations:** 1 Key Laboratory of South China Agricultural Plant Molecular Analysis and Genetic Improvement & Guangdong Provincial Key Laboratory of Applied Botany, South China Botanical Garden, Chinese Academy of Sciences, Guangzhou, China; 2 Center of Economic Botany, Core Botanical Gardens, Chinese Academy of Sciences, Guangzhou, 510650 P.R. China; 3 College of Life Sciences, University of the Chinese Academy of Sciences, Beijing, 100039 P.R. China; 4 College of Resources and Environment, University of the Chinese Academy of Sciences, Beijing, 100039 P.R. China

**Keywords:** Abiotic, Drought, Salt

## Abstract

Late embryogenesis abundant (LEA) proteins belong to a large family that exists widely in plants and is mainly involved in desiccation processes during plant development or in the response to abiotic stresses. Here, we reported on an atypical *LEA* gene (*IpLEA*) related to salt tolerance from *Ipomoea pes-caprae* L. (Convolvulaceae). Sequence analysis revealed that IpLEA belongs to the LEA_2 (PF03168) group. IpLEA was shown to have a cytoplasmic localization pattern. Quantitative reverse transcription PCR analysis showed that *IpLEA* was widely expressed in different organs of the *I*. *pes-caprae* plants, and the expression levels increased following salt, osmotic, oxidative, freezing, and abscisic acid treatments. Analysis of the 1,495 bp promoter of *IpLEA* identified distinct *cis*-acting regulatory elements involved in abiotic stress. Induction of IpLEA improved *Escherichia coli* growth performance compared with the control under abiotic stresses. To further assess the function of *IpLEA* in plants, transgenic Arabidopsis plants overexpressing *IpLEA* were generated. The *IpLEA*-overexpressing Arabidopsis seedlings and adult plants showed higher tolerance to salt and drought stress than the wild-type. The transgenic plants also showed higher oxidative stress tolerance than the wild-type Arabidopsis. Furthermore, the expression patterns of a series of stress-responsive genes were affected. The results indicate that *IpLEA* is involved in the plant response to salt and drought, probably by mediating water homeostasis or by acting as a reactive oxygen species scavenger, thereby influencing physiological processes under various abiotic stresses in microorganisms and plants.

## Introduction

During their life cycle, plants are constantly exposed to various adversities, including abiotic and biotic stresses, which affect their development and growth^1^. The exposure to these forms of stresses do negatively results into low crop yield and quality. Of these challenges, water deficit is a pervasive cellular issue confronting both aquatic and terrestrial plants^2^. There are several types of adversity that could result in cellular water deficit in plants, including freezing, drought, and high salinity^1,2^. In the evolutionary adaptation process of plants to these adversities, plant cells have developed a series of physiological and molecular mechanisms to reduce or relieve injures caused by water deficit, including structural changes and alterations to molecular synthesis and metabolism^3^. A variety of protective proteins, chaperones, reactive oxygen species (ROS)-detoxification proteins, and transcription factors (TFs) are supposed to mediate the protective responses against external stresses to maintain cellular metabolic and structural integrity, as well as to ensure that the plants can complete their growth and development cycle^1^. Late embryogenesis abundant proteins (LEA proteins), as a sort of protective protein, are ubiquitous hydrophilic proteins in plants and are mainly involved in protection to desiccation during seed dehydration, or vegetative tissues under stress conditions by acting as cellular dewatering protectants^4^.

The first plant *LEA* mRNA was identified from cotton (*Gossypium hirsutum*) seeds and accumulated during late embryogenesis^5^. Since this discovery, numerous *LEA* members have been extensively characterized over the past three decades and are not only limited to plants^2^. Many reports have indicated that the accumulation of LEA proteins or mRNA is closely associated with the desiccation tolerance of plant tissues, but their specific function remains unknown, particularly in extremophytes. In general, these types of proteins are composed of a high percentage of charged amino acid residues as well as glycine (Gly) or other small amino acids, such as alanine (Ala), serine (Ser), and threonine (Thr), but lack or only contain small amounts of tryptophan (Trp) and cysteine (Cys) residues^6^, lead to their classification as ‘hydrophilic’ due to their high hydrophilicity^6,7^. LEA proteins are predicted to be intrinsically disordered in the fully hydrated state and then may become folded in the dry state and acquire α-helical structures. The ordered LEA proteins under dehydration possibly bind to enzymes, membranes, DNA/RNA, water, or ions, and ROS, thereby stabilizing proteins and membranes under abiotic stress^8^, as well as providing aid in the formation and stability of an intracellular glassy state that is indispensable for the survival of plant propagules in the dry state^9^.

LEA genes are highly represented in plant genomes; for instance, 51 *LEAs* have been documented in *Arabidopsis thaliana*^10^, 34 *LEAs* in rice^11^, 27 *LEAs* in tomato^12^, 72 *LEAs* in sweet orange^13^, 23 *LEAs* in pine^14^, 108 *LEAs* in canola^15^, 23 *LEAs* in bamboo^16^, 17 *LEAs* in *Dendrobium officinale* (Orchidaceae)^17^, 26 *LEA* members in cassava^18^, and over 100 *LEA* members in three gossypium ecotypes^19^. Plant LEA proteins can be grouped into eight families in the PFAM database according to the eight PFAM motifs (PF03760, PF03168, PF03242, PF02987, PF00477, PF00257, PF04927, and PF10714)^20^. Different LEA members appear to have specific subcellular distributions, such as in the cytosol, mitochondria, chloroplasts, endoplasmic reticulum, and nucleus^10^. This diversity in LEA proteins in plants indicates that their physiological function and modes of action under abiotic stress may be universal. However, their individual roles have not been well-characterized, especially in the halophytes of Convolvulaceae.

Although over 1000 LEA sequences have been gathered in the dedicated LEA protein database (LEAPDB; http://forge.info.univ-angers.fr/gh/Leadb/index.php)^20^, only a few LEAs have been functionally characterized, and thus the biological roles of most members of the LEA family still remain unclear. Many studies on LEA proteins have focused on the protein structure, subcellular localization, transcriptional regulation, and acting as molecular chaperone or protective protein^8^. Some reports demonstrated that the heterologous overexpression of *LEA*s could improve stress tolerance in transgenic plants, yeast, and bacteria. For example, *Thellungiella salsuginea* (formerly known as *Eutrema salsugineum*) is a valuable halophytic genetic model plant, and the ectopic expression of *TsLEA1* from *T*. *salsuginea* confers salt-tolerance in yeast and Arabidopsis^21^. *Salvia miltiorrhiza* is a well-known traditional Chinese herbal plant with strong environmental adaptability, and the overexpression of *SmLEA1* and *SmLEA2* enhances salt and drought tolerance in *Escherichia coli* and *S*. *miltiorrhiza*^22^. Foxtail millet is a remarkably drought-resistant plant, and the expression of a novel atypical LEA gene *SiLEA14* in foxtail millet and Arabidopsis demonstrably improved the salt and drought tolerance of transgenic plants^23^.

*Ipomoea pes-caprae* (Convolvulaceae), as a typical marginal-marine halophytic plant with a high level of nutrient utilization efficiency, is widely distributed on beaches or islands in tropical and sub-tropical regions and provides one of the best known examples of oceanic dispersal^24^. This plant has strong environmental adaptability and can be used as an ecological ‘green shelter’ in sand fixation, wind resistance, landscape greening, and ecological restoration in tropical and subtropical coral islands and coastal zones^25^. It is well-known that *I*. *pes-caprae* possesses remarkable salinity and drought resistance, and therefore can be considered as a good germplasm for the characterization of salt/drought-tolerance-related genetic resources. However, the molecular mechanisms underlying these tolerances are not well-defined.

We previously generated a cDNA library from salt-treated *I*. *pes-caprae* seedlings, including both the aerial parts and roots, using a pYES-DEST52 shuttle vector, and described a series of salt stress-related genes from this library^26^. Of these, a full-length cDNA encoding a LEA protein (*IpSR26*, *IpLEA*, GenBank Accession No.: MF680612) was further characterized in the present study. The overexpression of *IpLEA* resulted in enhanced resistance to abiotic stresses in *E*. *coli* and Arabidopsis. The *IpLEA* promoter mediated the remarkable induction of *β-glucuronidase* (*GUS*) expression in transgenic Arabidopsis under various stresses. *Cis*-acting regulatory elements in the *IpLEA* promoter were also predicted, and promoter-driven GUS expression was detected in transgenic Arabidopsis. These data reveal the potential application of *IpLEA* in the genetic engineering of plants with elevated salt or drought tolerance.

## Materials and Methods

### Plant materials, growth conditions, and stress treatments

The *I*. *pes-caprae* seeds and plants were gathered from beaches in Zhuhai City (22°16′25.37″N, 113°34′18.00″E), China. The seeds germination, seedling planting and stress or hormone treatment of *I*. *pes-caprae* was performed as previously described^27,28^. In brief, the seedlings with 4–6 true leaves were used for stress treatment assays to assess the expression patterns of *IpLEA*. Subsequently, salt (300 mM NaCl), simulated drought or dehydration (300 mM mannitol), oxidative (0.1 mM methyl viologen, MV), and cold (0 °C) stresses and abscisic acid (ABA) treatment (0.1 mM) were applied to the *I*. *pes-caprae* seedlings to detect the expression pattern of *IpLEA*.

*Arabidopsis thaliana* (ecotype Columbia) plants used for the ectopic overexpression experiments were grown on solid Murashige and Skoog (MS) plates for about 10 d before being transferred into nutrient solution-soaked vermiculite as potted medium. All of Arabidopsis plants, including germinating seeds and seedlings, were put in a controlled environment greenhouse at 22 °C, with a 16-h light/8-h darkness photoperiod, a light intensity of 120 mmol m^−2^ s^−1^, and 60–80% relative humidity. Three T3 homozygous transgenic lines were picked out and confirmed by PCR, and then were accessed for further phenotypic analysis as previously described^27,28^. To identify the expression patterns of the salt/drought stress response genes in Arabidopsis, transgenic lines and WT (3-week-old) seedlings planted in vermiculite were immersed in 200 mM NaCl or 300 mM mannitol solutions for 24 h, following which the leaves were harvested for RNA isolation.

### Cloning of *IpLEA* cDNA

A full-length cDNA library from *I*. *pes-caprae* was constructed and screened with a FOX (Full‐length cDNA Over‐eXpressing) gene hunting system using a yeast salt sensitive mutant (AXT3) complementary assay approach^26^. Thereafter, a full-length cDNA (*IpSR26*) encoding a LEA protein that could rescue the phenotype of AXT3 was selected for further investigation.

### Bioinformatics analysis of the IpLEA

The full-length LEA protein cDNA sequence (GenBank accession no.: MF680612) was translated by the online ORFfinder translate tool (https://www.ncbi.nlm.nih.gov/orffinder/). Two LEA_2 motifs (PF03168) were identified through the online NCBI blastp program (https://blast.ncbi.nlm.nih.gov/Blast.cgi?PROGRAM=blastp&PAGE_TYPE=BlastSearch&LINK_LOC=blasthome). MEGA 6 was used for the protein homology comparisons. The grand average of hydropathy (GRAVY) and other physical and chemical properties for IpLEA were predicted with the ExPaSy program (http://www.expasy.org/tools), and the 3D structural diagram of IpLEA was predicted by PHYRE^2^ (http://www.sbg.bio.ic.ac.uk/phyre2/html/page.cgi?id=index).

The amino acid sequence of IpLEA was aligned with known plant LEAs by ClustalW software (http://clustalw.ddbj.nig.ac.jp/). The amino acid sequences used were as follows: InLEA from *Ipomoea nil* (NCBI accession no.: XP_019174801), SmLEA2 from *S*. *miltiorrhiza* (NCBI accession no.: ADX9850), and Arabidopsis LEA_2 member (At2g44060.1).

### Isolation and functional analysis of IpLEA’s promoter region

The genomic DNA sequence of *IpLEA* was obtained by PCR amplification with primer pair IpLEAF and IpLEAR (Table S1). The isolation of *I*. *pes-caprae* genomic DNA and PCR were performed according to our previous study^27^. The 5′ flanking region upstream of the translation start codon (promoter sequence) of *IpLEA* was amplified from *I*. *pes-caprae* genomic DNA through genome walking using a Genome Walking Kit (Takara, Dalian) according to the manufacturer’s instructions. Three gene-specific primers (IpLEASP1, IpLEASP2, and IpLEASP3, Table S1) were adopted in the nested PCR process. Finally, the purified PCR products for putative *IpLEA*’s promoter region were then recovered from the agarose gel electrophoresis and ligated into a pGEM T-vector (Promega, Shanghai) and sequenced. The putative *cis*-acting elements of this promoter region (IpLEA-PRO) were analyzed using the online tool PlantCARE (http://bioinformatics.psb.ugent.be/webtools/plantcare/html/)^29^.

### Expression of IpLEA protein and assay of protective role of IpLEA in *E. coli*

The *IpLEA* CDS was amplified with the primers IpLEAEPF and IpLEAEPR (Table S1), and then PCR fragments were subsequently inserted into the *Bam*HI site of pET 28a, following the His-tag with the in-fusion technique (BD In-Fusion PCR cloning Kit, Takara), yielding the recombinant plasmid IpLEA-pET 28a. The recombinant plasmid and pET 28a (as a negative control) were then transformed into *E*. *coli Rossetta* (DE3). A single colony was then inoculated in liquid Luria-Bertani (LB) medium (containing 50 mg mL^−1^ kanamycin) and allowed to grow overnight at 37 °C with constant shaking at 200 rpm. Inoculum (1%) from the overnight grown culture was added to fresh LB medium (100 mL, containing 50 mg mL^−1^ kanamycin) and allowed to grow at 37 °C with constant shaking at 200 rpm. The isopropyl β-D-thiogalactopyranoside (IPTG)-induced expression of His-tag IpLEA was conducted and confirmed by 12% sodium dodecyl sulfate polyacrylamide gel electrophoresis (SDS PAGE), as previously described^27^.

A spot assay was performed to test the stress tolerance of the recombinant *E*. *coli*, with three replicates for each sample, basically according to our previous reports^27,28^. To evaluate salt, osmotic, and H_2_O_2_ stresses, cell cultures of *E*. *coli* containing pET 28a/IpLEA-pET 28a were adjusted to OD600 = 1.0 and then serially diluted (to 1:10, 1:100, and 1:1000). Two microliters of each sample was spotted onto the LB plates containing 0.2 mM IPTG and the stress treatment (5% NaCl, 1.5 M sorbitol, or 2 mM H_2_O_2_). For the drought test, 10 μL OD-adjusted cell cultures in tubes were immediately placed in a 40 °C drying oven where they were maintained for 4 h. Then, the samples were added to 100 μL liquid LB medium and dissolved at 37 °C for 1 h to recover. The samples were then diluted and spotted onto LB plates with 0.2 mM IPTG. The plates were incubated at 37 °C for 12–16 h. The bacterial colonies were then counted (Colony-Forming Unit, CFU), and the differences were analyzed.

For the growth curve in liquid culture assay, 1 mL inoculum (OD600 value 1.0) was added to 10 mL LB medium (containing 0.2 mM IPTG) containing salt (3% or 4% NaCl), sorbitol (0.8 M or 1 M), or H_2_O_2_ (0.7 mM or 0.9 mM), and incubated at 37 °C with shaking (200 rpm). The aliquots were removed from each treatment every 2 h for 12 h and the absorbance (OD600) was measured. Abiotic stress (salt, osmotic, and anti-oxidative) tolerances were determined with respect to the control cultures (bacterial cells with vector controls).

### Subcellular localization analysis

The CDS of the *IpLEA* cDNA generated by PCR amplification (with primer pair IpLEAGF and IpLEAGR, Table S1) was inserted into the *Bam*HI site of the pUC/green fluorescent protein (GFP) vector to generate the recombinant plasmid IpLEA-pUC/GFP. After sequencing confirmation, the fusion construct and control (empty vector) vectors were co-transfected with another NLS-mCherry vector separately into protoplasts. GFP fluorescence was visualized using a confocal laser scanning microscope (LSM, 510 META, Zeiss, Germany).

### Quantitative reverse transcription (qRT)-PCR

qRT-PCR was performed basically according to our previously reports^27,28^, with a purpose of accessing assessing the function of *IpLEA*. In brief, total RNA was isolated from the different tissues of *I*. *pes-caprae* with HiPure Plant RNA kits (Magen, Guangzhou), and the cDNA was synthesized from the total RNA using TransScript One-Step gDNA Removal and cDNA Synthesis SuperMix (TransGen Biotech, Beijing) with Oligo(dT)_15_ primers according to the manufacturer’s instructions. The expression levels of *IpLEA* in various organs of the seedling and adult *I*. *pes-caprae* plants, including the seedling root, seedling leaf, bud, mature root, vine, mature leaf, flower bud, petal, and young seeds, at 7 d after pollination (DAP), were detected respectively. The *I*. *pes-caprae* seedling samples (roots, vines, and leaves) treated with salt, simulated drought or dehydration, oxidative stress and frost treatment (0 °C), and ABA were also assessed to examine the expression changes of *IpLEA*. All of the gene expression data obtained via qRT-PCR were normalized to the expression of *IpUBQ* (GenBank accession number: MF502417). The primers used for qRT-PCR are listed in Table S1.

For detecting the expression of antioxidation system-related genes (*CAT1*, *CSD1*, *APX1*, and *ERD5*) and abiotic stress-related genes (*ANAC19*, *NCED3*, *HAI2*, *RD26*, *RD29A*, and *RD29B*) in Arabidopsis (WT or transgenic plants), the total RNA was isolated from rosette leaves at different time points (with or without treatments), and cDNA synthesis was performed using the above procedure. The reference gene for the qRT-PCR was *ACT2* (At3g18780) in Arabidopsis. The primers used for qRT-PCR are listed in Table S1.

### DNA constructs and generation of transgenic plants

To construct the *IpLEA* promoter/GUS fusion products *IpLEA*-PRO/pBI101.2, the pGEM-T vector containing the 1.5 kb *IpLEA* promoter was used as template DNA to PCR amplify the full-length *IpLEA* promoter fragment. The primer pair IpLEAProF and IpLEAProR is listed in Table S1. To generate the recombinant vector for the overexpression assay in Arabidopsis, the CDS of the *IpLEA* cDNA was PCR-amplified using the primer pair IpLEAOXF and IpLEAOXR (Table S1). The PCR product was cloned into the *Bam*HI sites of the plant expression vectors pBI101.2 and pBIm^27,28^ to generate *IpLEA*-PRO/pBI101.2 (Fig. S1A) and *IpLEA*/pBIm (Fig. S1B), following the in-fusion technique (BD In-Fusion PCR cloning Kit, Takara Bio USA).

After sequencing confirmation, the constructs were transferred into *Agrobacterium tumefaciens* GV3101 and then transformed into Arabidopsis using the floral dip method. Seeds of the T1 and T2 generations were screened on MS agar medium containing 50 mg L^−1^ kanamycin. Positive transgenic plants were selected according to the segregation ratio (resistant: sensitive = 3: 1) and confirmed by genomic PCR with the primer pairs IpLEAOXF/IpLEAOXR and IpLEAProF/IpLEAProR. Finally, T3 homozygous transgenic and WT seeds were germinated and used in the plant tolerance assays, or the T3 homozygous transgenic seedlings were used in the GUS staining assay.

### Histochemical GUS staining and expression analysis of *GUS* under *IpLEA*-PRO control

We subjected the T3 seedlings of the transgenic Arabidopsis plants (containing the *IpLEA* promoter driven by *GUS*) to GUS staining according to Jefferson *et al*.^30^. T3 seedlings of the transgenic Arabidopsis plants were treated with salt (200 mM NaCl) or osmotic stress (300 mM mannitol) for 24 h, with the untreated transgenic Arabidopsis plants as controls. The GUS-positive plant tissues were examined using a light microscope at low magnification and photographed. To further identify the characteristics of the *IpLEA* promoter, *GUS* expression in the transgenic Arabidopsis plants driven by the upstream 1,495 bp of ATG was also evaluated using qRT-PCR. The primer pairs for *GUS* and *AtAct2* are listed in Table S1. The provided images and data of the GUS-stained tissues and *GUS* expression analysis represent the typical results of at least three independent transgenic lines.

### Abiotic stress tolerance assays in transgenic Arabidopsis

The seed germination rate of the *IpLEA* transgenic Arabidopsis (*# 1*, *# 9*, and *# 11*) was detected under NaCl (175 mM, 200 mM, and 225 mM) and mannitol (200 mM, 300 mM, and 400 mM) stress to detect the effect of the overexpression of *IpLEA* on improving the salt/osmotic tolerance of transgenic Arabidopsis seeds during germination. Additionally, root length was also calculated to evaluate the influence of the overexpression of *IpLEA* on transgenic Arabidopsis seedlings under abiotic stress (100 mM, 150 mM, and 200 mM NaCl for salt stress or 200 mM, 300 mM, and 400 mM mannitol for osmotic stress). WT Arabidopsis and MS medium were used as control. The seed germination and seedling growth experiments were both performed on MS plates with or without stress factors, in the same greenhouse environment as Arabidopsis plants growing.

Salt and drought tolerance assays were also performed on transgenic Arabidopsis adult plants according to our previous study^27,28^. Both WT and transgenic seeds (*# 1*, *# 9*, and *# 11*) were grown on MS medium. Ten-day-old seedlings were planted in sieve-like square pots filled with nutrient solution soaked vermiculite. Thirty plants of each genotype were cultured in greenhouse as described above without watering for another 20 d to make sure seedlings growing up, and the water content of vermiculite in planting pots reduced but has not caused plants drought stress. The plants were then subjected to the following assays. For the drought tolerance assays, WT and transgenic plants (*# 1*, *# 9*, and *# 11*) were maintained under continuous drought conditions for 9 d and then re-watered for 7 d. For the salt tolerance assays, plants of each genotype (*# 1*, *# 9*, *# 11*, and WT) were planted in sieve-like pots and well-watered as described for the drought tolerance treatment. Water was withheld for 20 d prior to irrigation with NaCl solution (150 mM and 200 mM) from the bottom of the plants. When the vermiculite was completely saturated with salt water, the NaCl solution was removed from the bottom the planting tray and the plants were cultured without watering. The plants grew in the salt-saturated vermiculite for another 23 d and were took pictures.

For the oxidative stress analyses of the transgenic overexpression lines and WT plants, the seed germinnation, seedling growth, and adult plant of *IpLEA OXs* (*# 1*, *# 9*, and *# 11*) and WT Arabidopsis were also assessed under H_2_O_2_ or MV challenges in similar method with the salt/osmotic tolerance of Arabidopsis plants. In brief, for the seed germination rate analysis on MS plates, the concentrations of H_2_O_2_ were 0 (as a control), 3.5 mM, 4 mM, and 4.5 mM respectively; for the seedling root length on MS plates, the concentrations of H_2_O_2_ were 0 (as a control), 2.5 mM, 3 mM, 3.5 mM, and 4 mM respectively. In addition, the 30-day-old seedlings of *IpLEA OXs* (*# 1*, *# 9*, and *# 11*) and WT Arabidopsis adult plants grown in vermiculite were sprayed evenly with 20 μM methyl viologen (MV) (spraying was repeated ten times each plant and make sure each leaf of Arabidopsis plants evenly covering MV solution, one spray ≈ 100 μL), after which the plants were cultured with necessary watering for 18 d. The phenotype was recorded.

### ROS staining assay

Rosette leaves were collected from 3-week-old seedlings (*#1*, *#9*, *#11* and WT) growing in the soil, following which the leafstalks were immersed in 200 mM NaCl or 300 mM mannitol solution for 24 h. *In situ* detection of H_2_O_2_ and O_2_^−^ was determined using vacuum-infiltrating with 1 mg/mL nitro-blue tetrazolium (NBT) or 1 mg/mL 3.3′-diaminobenzidine (DAB) solution, respectively, for 12 h, followed by clearing in 75% ethanol^27,28^.

### Statistical analysis

All of the experiments were repeated three times in independent experiments, and the data shown are the mean ± standard deviation (SD). In this research, the data were subjected to Student’s *t* test analyses using Microsoft Excel software. Asterisks indicate significant differences based on Student’s *t* test between the WT and transgenic lines (*P < 0.05; **P < 0.01).

## Results

### *IpLEA* encodes a LEA_2 type LEA protein

**Figure 1:**
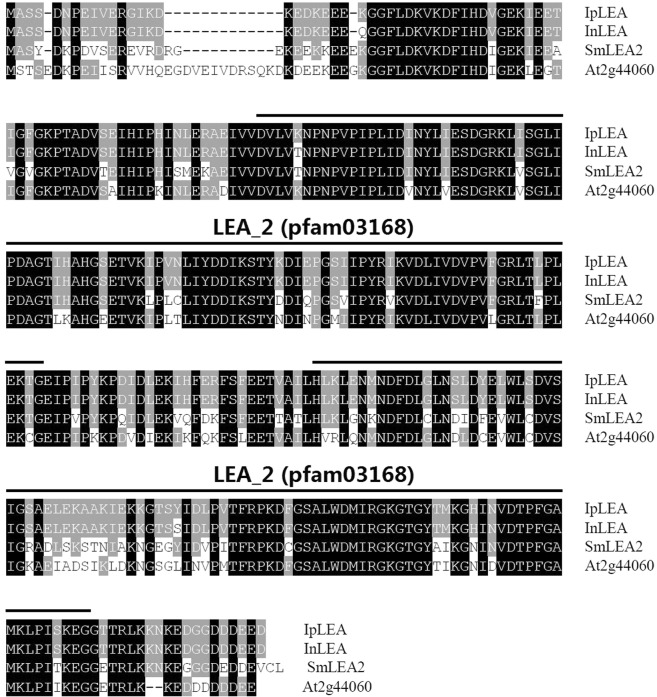
Multiple sequence alignment of IpLEA with its homologous sequences, including *I*. *nil* InLEA (XP_019174801.1), *S*. *miltiorrhiza* SmLEA2 (ADX9850.1), and Arabidopsis LEA (At2g44060.1). The amino acid sequences of two LEA_2 motifs (PF03168) are indicated with lines.

The *IpLEA* cDNA was determined from a cDNA library of *I*. *pes-caprae* by FOX-hunting technique^26^. The cDNA sequence of *IpLEA* encodes a polypeptide of 313 amino acid residues with a 61 bp 5′ untranslated region (UTR) and a 390 bp 3′ UTR (including a polyA tail). The physical and chemical properties of IpLEA protein were summarized in Table S2. Generally, as IpLEA being a hydrophilin^6^ and IDP^8^, the GRAVY value and the instability index of IpLEA is −0.373 and 29.09 respectively (ExPASy ProtParam, http://web.expasy.org/protparam/). It is rich in hydrophilic amino acids, such as aspartic acid (Asp) (D, 9.9%), glutamic acid (Glu) (E, 9.58%), histidine (His) (H, 8.37%), lysine (Lys) (K, 9.58%), Ser (S, 5.11%), and Thr (T, 4.79%), but contains low quantities of hydrophobic amino acids, such as methionine (Met) (M, 1.6%), phenylalanine (Phe) (F, 2.33%), Pro (P, 1.39%), and Trp (W, 0.64%), and lacks Cys (C) and glutamine (Gln) (Q). A blastp search of the IpLEA protein in NCBI revealed that it contains two “LEA_2” motifs (PF03186) (Fig. 1), which were classified into subgroup LEA_2 according to Hundertmark’s classification of LEA proteins^31^. IpLEA is highly homologous to the LEA protein InLEA from *Ipomoea nil*, SmLEA2 from *S*. *miltiorrhiza*, and LEA_2 member At2g44060 from *A*. *thaliana* (Fig. 1). IpLEA 3D predication indicated that IpLEA contains several α-helixes and β-sheets and presented certain secondary structure (Fig. S2).

### Analysis for promoter region of *IpLEA*

**Figure 2:**
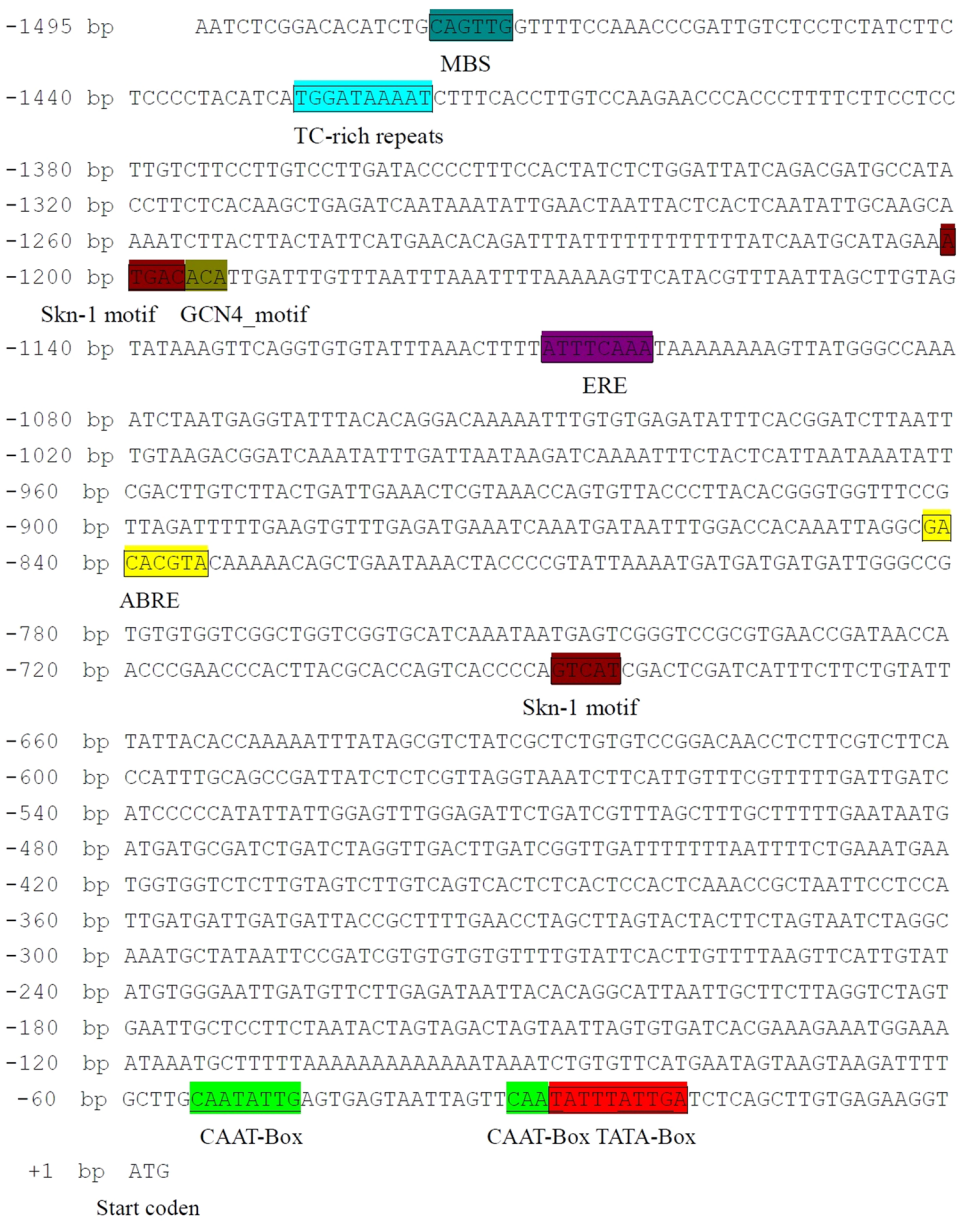
Bioinformatics analyses of the DNA sequences of the *IpLEA* promoter. The initiation codon ATG for IpLEA protein is indicated with +1, and the putative *cis*-acting elements are shown inside boxes. The numbers on the left are the genomic DNA positions of each coding sequence. The partially putative *cis*-elements in the *IpLEA* promoter are marked with different colors.

The *IpLEA* genomic sequence harbors just one exon (without an intron) in its coding region. Based on the genomic region of the *IpLEA* sequence and the Genome Walking Kit manual, a 1,495 bp promoter region sequence was obtained for *IpLEA*. The possible *cis*-elements were predicted by the online program PlantCARE, which indicated that a number of potential *cis*-acting elements that might be involved in binding some specific TFs were present (Fig. 2), and these putative elements are classified in Table 1. A putative TATA-Box motif (tcTATAAAta) was detected at −29 bp upstream of the transcription start site (TSS), and four CAAT-Box motifs (CAAT) were located at −24 bp, −32 bp, −51 bp, and −55 bp upstream of TSS. Excluding these two core promoter elements, there were still 10 predicted *cis*-elements, including an ABA response element (ABRE, −842 bp), a binding site for myeloblastosis (MYB) transcription factors (MBS, −1478 bp), and a TC-rich repeats (−1428 bp) that were identified in the *IpLEA* promoter. The ABRE, MBS, and TC-rich repeats elements are supposed to bind specific TFs to regulate gene expression under some distinctive environmental factors or developmental signals. The ethylene-responsive element (ERE) and two endosperm activating expression *cis*-elements (Skn-1 motif or GCN4_motif) were also found at −1,110 bp, −689 bp, and −1,200 bp (Fig. 2). Here the putative seed-specific *cis*-elements showed relatively higher frequency (3 times, two Skn-1 motifs and one GCN4_motif) than other stress- or hormone- regulated elements, which might further implied the mRNA of *IpLEA* accumulating during the late embryogenesis of *I*. *pes-caprae* seed development. And also, the light regulated *cis*-elements can be found in the promoter region with high frequency (Box I, Sp1, and TCT-motif, Table 1), which indicated that light could affect the transcription of *IpLEA*. Although the exact roles of these *cis*-acting elements binding to specific TFs need be further confirmed by electrophoretic mobility shift assay (EMSA), at least, the presence of these *cis*-elements in the *IpLEA* promoter region indicated that the transcription of *IpLEA* might be regulated dynamically by binding the TFs, and that *IpLEA* was probably involved in some abiotic stress responses by transcriptional regulation.

**Table 1:** Regulatory elements identified in the promoter regions of *IpLEA*.

No	*cis*-Elements	Location (upstream of ATG)	Core sequence (5′ to 3′)	Putative function (species)
1	TATA-Box	−29	tcTATAAAta	Core promoter element around −30 of transcription (*Nicotiana tabacum*)
2	CAAT-Box	−24, −32, −51, −55	CAAT	Commom *cis*-acting element in promoter and enhancer regions around −80 to100 of transcription (*Hordeum vulgare*)
3	ABRE	−842	TACGTGTC	*cis*-acting element involved in the ABA responsiveness (*Arabidopsis thaliana*)
4	MBS	−1,478	CAACTG	MYB binding site involved in drought-inducibility (*Arabidopsis thaliana*)
5	TC-rich repeats	−1,428	ATTTTCTCCA	*cis*-acting element involved in defense and stress responsiveness (*Nicotiana tabacum*)
6	Skn-1 motif	−689, −1,201	GTCAT	*cis*-acting regulatory element required for endosperm expression (*Oryza sativa*)
7	GCN4_motif	−1,200	TGTGTCA	*cis* -regulatory element involved in endosperm expression (*Oryza sativa*)
8	ERE	−1,110	ATTTCAAA	Ethylene-responsive element (*Dianthus caryophyllus*)
9	G-box	−840	CACGTA	*cis*-acting regulatory element involved in light responsiveness (*Antirrhinum majus*, *Daucus carota*)
10	Box I	−1,109	TTTCAAA	Light responsive element (*Pisum sativum*)
11	Sp1	−912, −1,398	CC(G/A)CCC	Light responsive element (*Zea mays*)
12	TCT-motif	−70, −953, −1,019, −1,257	TCTTAC	Part of a light responsive element (*Arabidopsis thaliana*)

### The accumulation of IpLEA protein improves abiotic stress tolerance in *E. coli*

**Figure 3:**
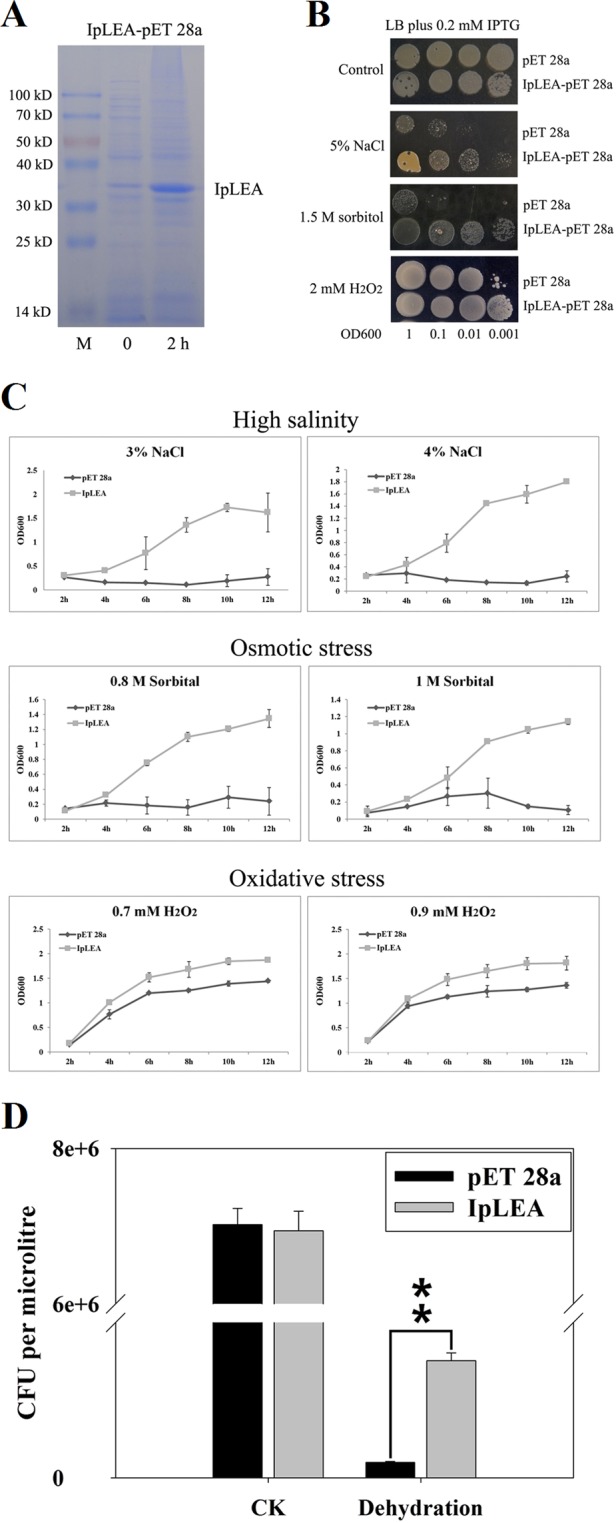
Functional analysis of the accumulation of the His-tag IpLEA for salt, osmotic/drought, and H_2_O_2_ tolerance in *E*. *coli*. (**A**) Induced expression of the IpLEA (IpLEA-pET 28a) protein in *E*. *coli*. 0 and 2 h: the IPTG induction times, respectively. (**B**) The growth performance of *E*. *coli* DE3 (pET 28a, upper)/(IpLEA-pET 28a, lower) on LB plates containing stress factors. Control (top): LB medium; 5% NaCl: LB medium containing 5% NaCl; 1.5 M Sorbitol: LB medium containing 1.5 M sorbitol; 2 mM H_2_O_2_: LB medium containing 2 mM H_2_O_2_. The cell cultures were adjusted to OD600 = 1 and were then diluted serially (1:10, 1:100, and 1:1000). Two microliters of each sample was spotted onto the LB plates containing 0.2 mM IPTG. (**C**) Growth kinetics of *E*. *coli* transformed with pET 28a (control) and IpLEA-pET 28a. Cells were grown until an optical density of 1.0 was reached at 600 nm, after which 0.2 mM IPTG was added and incubated for 2 h at 37°C. Subsequently, the cells were transferred to fresh LB medium (1:100, plus 0.2 mM IPTG) supplied with different concentrations of NaCl (3% or 4%), sorbitol (0.8 M or 1 M), or H_2_O_2_ (0.7 mM or 0.9 mM). The bacteria were cultured at 37 °C and 200 rpm. The OD600 values were measured every 2 h to evaluate the growth conditions; (**D**) cell viability related to CFU before (control) and after desiccation (40 °C for 4 h). Error bars indicate the ± SD based on three replicates. Asterisks indicate significant differences from the control (Student’s *t* test, **P < 0.01).

The recombinant IpLEA protein was induced in *E*. *coli* by IPTG to further confirm that the accumulation of IpLEA could improve abiotic stress tolerance in the bacterium. As shown in Fig. 3A, the His-tag IpLEA protein possessed a molecular weight of about 35 kDa, which is identical to the expected size. This result indicated that His-tag IpLEA protein has greatly accumulated in the induced *E*. *coli* cells.

After IpLEA’s induction, the *E*. *coli* cells in liquid LB medium were spotted on LB plates with stress factors (5% NaCl, 1.5 M sorbitol, or 2 mM H_2_O_2_, Fig. 3B), or were transferred into liquid LB medium with stress factors (3% or 4% NaCl, 0.8 M or 1 M sorbitol, and 0.7 or 0.9 mM H_2_O_2_, Fig. 3C), with the purpose of assessment the effect of IpLEA accumulation for *E*. *coli* cells’ stress tolerance. As we can see from Fig. 3B, the His-tag IpLEA accumulating cells showed much better growing status than *E*. *coli* control cells (with pET 28a). When assayed with the liquid culture assay, the growth of *E*. *coli* cells expressing His-tag IpLEA increased rapidly with time compared to the *E*. *coli* cells expressing the empty vector (pET 28a) control (Fig. 3C). Meanwhile, we also performed the dehydration-tolerance of bacteria expressing His-tag IpLEA. After desiccation and rehydration treatment, bacteria with accumulating His-tag IpLEA had a higher survival rate (six times higher) than control cells (pET 28a, Fig. 3D). Our result suggests that IpLEA expression in *E*. *coli* improved its survival capacity after desiccation.

### IpLEA is localized in cytoplasm in plant cells

**Figure 4:**
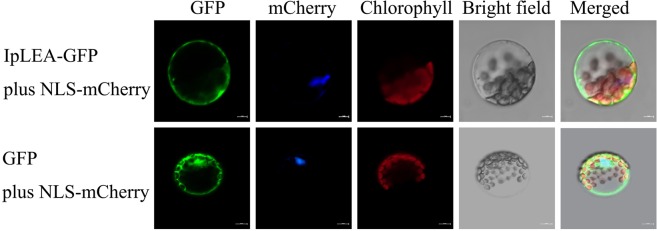
Subcellular localization of the IpLEA protein. Arabidopsis protoplasts expressing 35 S::*GFP* (**A**) and 35 S::*IpLEA-GFP* (**B**) fusion proteins observed under a laser scanning confocal microscope.

The subcellular localization of IpLEA was determined by an *in vivo* targeting experiment in which IpLEA-fused soluble enhanced GFP was transiently expressed in Arabidopsis mesophyll protoplasts. The IpLEA-GFP (Fig. 4, upper row) showed a similar localization pattern with the control GFP (Fig. 4, lower row), while with an obvious absence from the nucleus with NLS-mCherry as control (Fig. 4). Our results indicated that IpLEA is predominantly localized to the cytoplasm. In addition, we also predicted the subcellular localization values of IpLEA using the online program WoLF PSORT (http://wolfpsort.org/), which showed that IpLEA has a relatively higher likelihood for distribution in the cytoplasm (with a high value of 9 for the cytoplasm but lower values of 3 and 1 for the cytoskeleton and nucleus, respectively). Furthermore, the Plant-PLoc program (http://www.csbio.sjtu.edu.cn/bioinf/plant/) also indicated that IpLEA was mainly located in the cytoplasm.

### Expression pattern of *IpLEA* in *I. pes-caprae*

**Figure 5:**
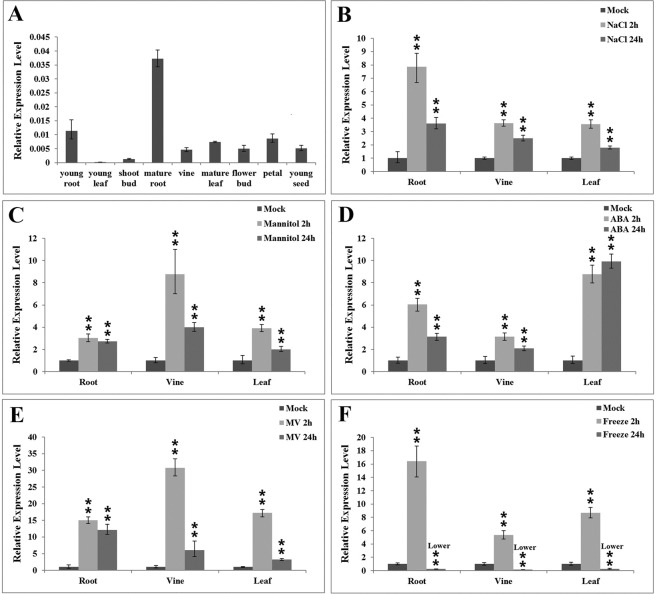
Expression profiles of *IpLEA* among *I*. *pes-caprae* tissues. (**A**) Differential expression of IpLEA in various tissues (young root, young leaf, shoot bud, mature root, vine, mature leaf, flower bud, petal, young seed). Time-course expression patterns of *IpLEA* in response to different abiotic stresses: high salinity (**B**), osmotic stress (**C**), ABA (**D**), MV (**E**) and cold treatment (**F**). Error bars indicate the ± SD based on three replicates. Asterisks indicate significant differences from the control (Student’s *t* test, *P < 0.05 and **P < 0.01).

To examine the expression pattern of *IpLEA* in *I*. *pes-caprae*, qRT-PCR was performed with total RNA extracted from various *I*. *pes-caprae* plant tissues. Our results revealed that *IpLEA* was expressed widely in most *I*. *pes-caprae* tissues (Fig. 5A). The highest transcription level of *IpLEA* was detected in the mature root, vine, and leaf, and the young root and flower petal also showed high expression of *IpLEA*. Conversely, *IpLEA* was weakly expressed in the tissues/cells that were rapidly growing, dividing, and metabolizing, such as young leaves and shoot buds.

To study the expression changes of *IpLEA* under abiotic stresses, the transcription of *IpLEA* was analyzed by qRT-PCR in *I*. *pes-caprae* seedling roots, vines, and leaves. When challenged by 300 mM NaCl, the transcript level of *IpLEA* showed the most obvious increase of over 7 fold in the root, and lesser increase in the vine and leaf tissues (Fig. 5B). Under dehydration stress (300 mM mannitol, simulating water-deficit or drought), the entire *I*. *pes-caprae* seedlings exhibited increased *IpLEA* expression patterns, while in the vine, the level of induction reached almost 10 fold (Fig. 5C). ABA also induced the expression of *IpLEA* in the *I*. *pes-caprae* seedlings, particularly in the leaf, peaking at 10-fold (Fig. 5D). MV (for oxidative stress) also rapidly and dramatically induced the expression of *IpLEA*, peaking at 2 h (Fig. 5E). Additionally, the expression changes of *IpLEA* under low temperature (0 °C) stress also showed obviously induced (Fig. 5F). Our results showed that cold treatment greatly and rapidly increased the transcription of *IpLEA* in a short time, while the expression seemed to be transiently, most probably due to a long time exposure to cold causing actual tissue damage in plant.

### Characterization of *IpLEA* promoter activity and assessment of the response of the promoter of *IpLEA* to abiotic stresses in transgenic Arabidopsis

**Figure 6:**
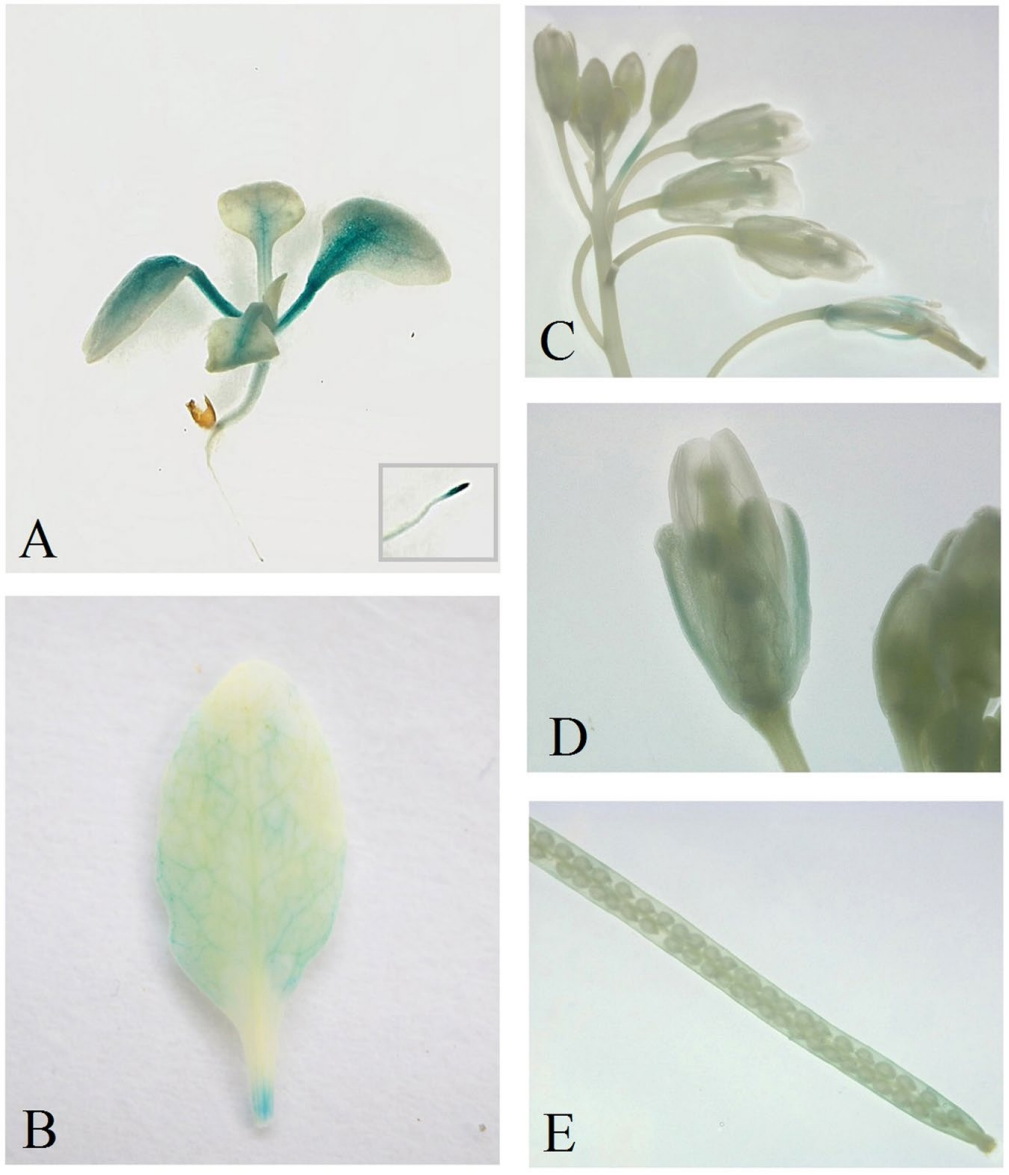
Histochemical GUS staining of transgenic Arabidopsis harboring the *IpLEA*-PRO::*GUS*. (**A**) Seedlings; (**B**) adult leaf; (**C**) inflorescence; (**D**) flower; (**E**) silique.

To test the activity of the promoter region, *IpLEA*-PRO*::GUS* (Fig. S1A) was transformed into Arabidopsis for preliminary analysis. GUS staining was examined in the T3 generation of the three transgenic lines. As shown in Fig. 6, the 2-week-old seedlings exhibited strong GUS staining in the cotyledons, emerging true leaves, and roots, and particularly in the root tips, while faint staining was observed in the hypocotyls (Fig. 6A). Blue precipitate was also appeared in the different parts of the adult plants, including the leaves, inflorescence stems, flowers, and siliques (Fig. 6B–E). The staining results verified that the *IpLEA* promoter was able to drive transgene (*GUS*) expression and was constitutively lowly expressed in transgenic Arabidopsis.

**Figure 7:**
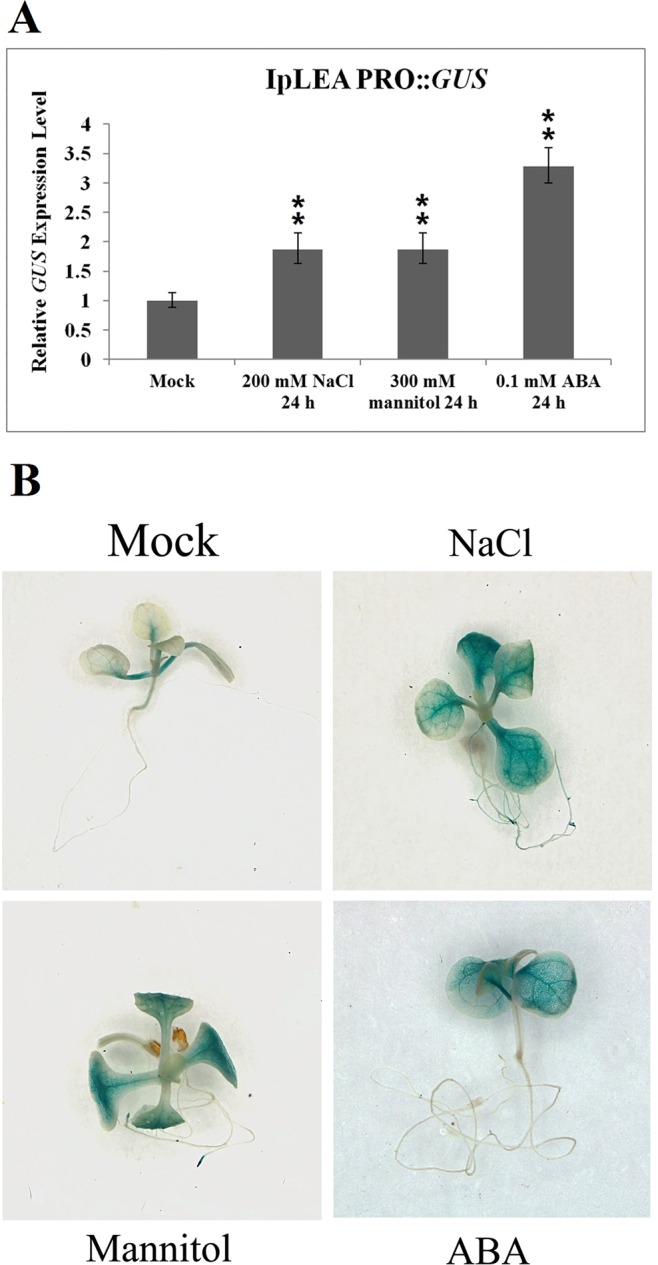
Expression analysis of the *GUS* gene and GUS staining in seedling transgenic Arabidopsis plants. (**A**) *GUS* expression under the control of *IpLEA*-PRO. Error bars indicate the ± SD based on three replicates. Asterisks indicate significant differences from the control (Student’s *t* test, **P < 0.01); (**B**) GUS staining of transgenic Arabidopsis seedlings containing the *IpLEA*-PRO::*GUS* construct under normal and stress conditions. NaCl (200 mM, 24 h); mannitol (300 mM, 24 h); ABA (0.1 mM, 24 h).

To further characterize the features of the cloned *IpLEA* promoter, we also evaluated the *GUS* gene expression pattern in transgenic Arabidopsis seedlings by qRT-PCR. As shown in Fig. 7A, under high salinity (200 mM NaCl), osmotic stress (300 mM mannitol), and ABA (0.1 mM ABA) treatments, the *GUS* gene exhibited an induced expression pattern, which indicated that even in Arabidopsis, the cloned *IpLEA* promoter region appeared to be a water-deficit/ABA-induced promoter (Fig. 7A). Furthermore, both the treatment and control seedlings were stained with GUS reaction mixture. The results also confirmed that *GUS* expression was obviously induced by water-deficit/ABA after 24 h of treatment (Fig. 7B). Our results demonstrated that the full-length *IpLEA* promoter sequences positively responded to drought/salt stress and ABA treatment.

### *IpLEA* exhibits tolerance to high salinity and drought stresses in plants

**Figure 8:**
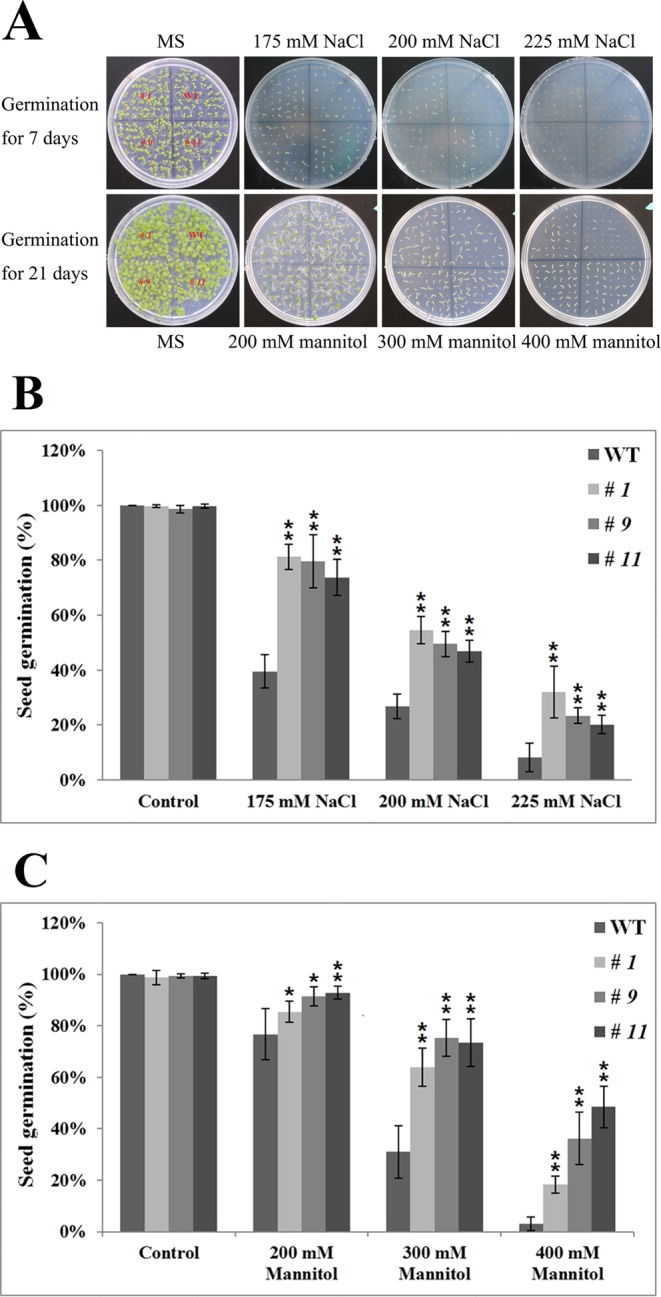
Osmotic and salt stress analyses of *IpLEA* transgenic *OX* lines and WT with respect to the seed germination rate. (**A**) Photographs of three transgenic lines (*# 1*, *# 9*, and *# 11*) and WT seeds germinated on MS medium or MS medium with NaCl (upper, 175 mM, 200 mM, and 225 mM) for 7 d or mannitol (lower, 200 mM, 300 mM, and 400 mM) for 21 d. Seed germination rates were calculated for the WT and transgenic lines under NaCl (**B**) and mannitol (**C**) stress after 7 d. Error bars indicate the ± SD based on three replicates. Asterisks indicate significant differences from the WT (control, Student’s *t* test, *P < 0.05 and **P < 0.01).

**Figure 9:**
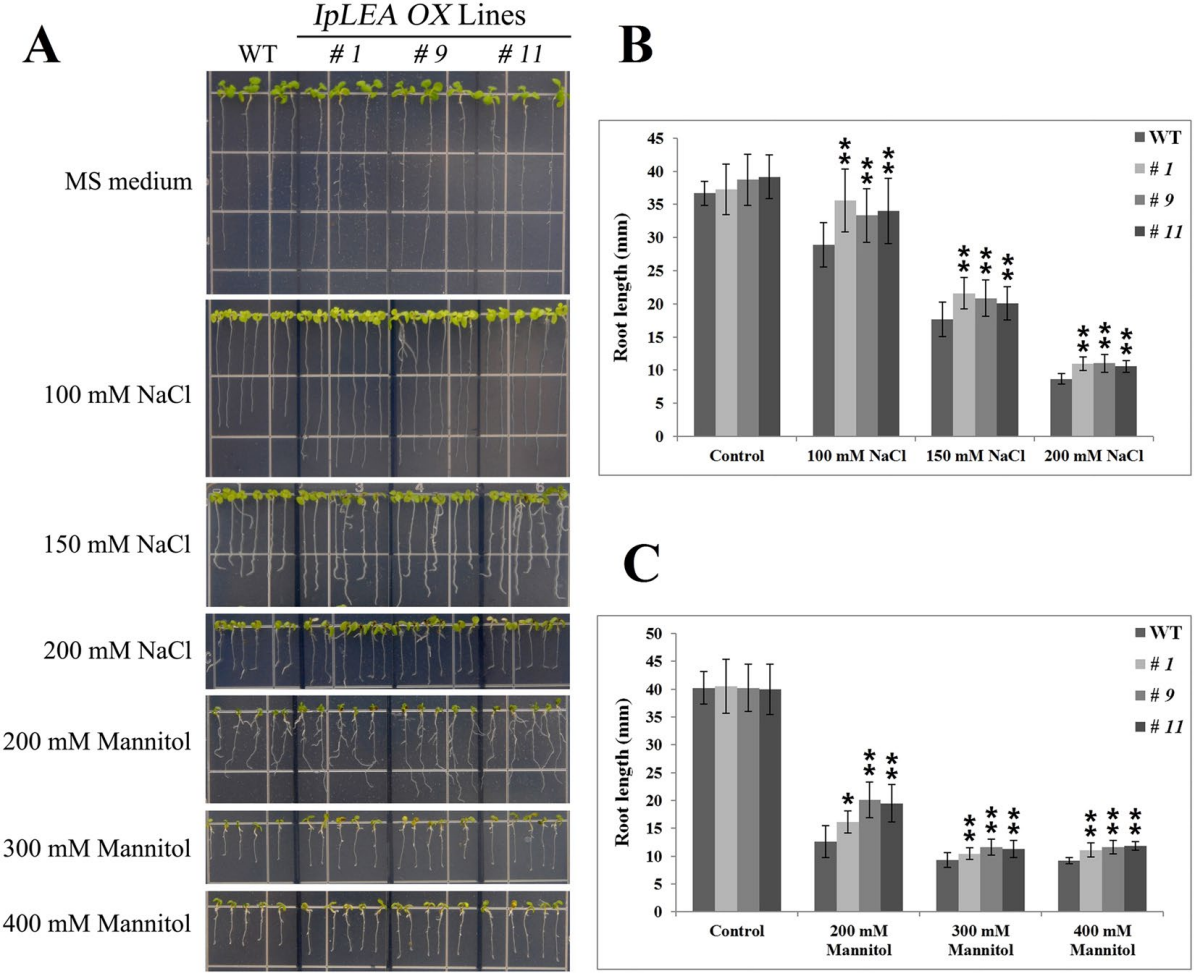
Osmotic and salt stress analyses of *IpLEA* transgenic *OX* lines (*# 1*, *# 9*, and *# 11*) and WT with respect to seedling root length. Four-day-old seedlings were transplanted to MS medium containing NaCl or mannitol and were then grown for 7 d before measuring the root length. (**A**) Photographs of *IpLEA* transgenic *OX* lines and WT seedlings on MS medium or MS medium with NaCl (right, 100 mM, 150 mM, and 200 mM) or mannitol (200 mM, 300 mM, and 400 mM); (**B**,**C**) Seedling root length of WT and *IpLEA* transgenic *OX* lines under NaCl (**B**) or mannitol (**C**) stress after 7 d. Error bars indicate the ± SD based on three replicates. Asterisks indicate significant differences from the WT (control, Student’s *t* test, *P < 0.05 and **P < 0.01).

The *IpLEA* gene was introduced into Arabidopsis in order to study its function in plants under the control of the 35 S promoter (Fig. S1B). Three transgenic homozygous lines (T3) were obtained by kanamycin screening. Three lines were selected for further investigation, which were designated as *IpLEA OX* lines *# 1*, *# 9*, and *# 11*, and then the DNA insertion and *IpLEA* expression were assessed by genomic DNA PCR/RT-PCR (Fig. S3A) and qRT-PCR (Fig. S3B). Accordingly, in the follow-up experiment, plant tolerance assays were also performed using these three lines. First, we examined germination and seedling growth in the WT and *IpLEA OX* lines under salt/osmotic stresses to elucidate the abiotic stress response in the *IpLEA* overexpression plants. Seeds from WT and *IpLEA OX* lines *# 1*, *# 9*, and *# 11* were germinated and grown on MS plates (as a control), and MS medium containing NaCl (175 mM, 200 mM, and 225 mM) or mannitol (200 mM, 300 mM, and 400 mM) to evaluate their stress tolerance. On the MS plates, the germination rate and seedling growth of the WT and transgenic lines showed no differences. However, in the presence of NaCl and mannitol, the seeds and seedlings (7 or 21 d after germination) from the transgenic line exhibited significantly higher germination rates (Fig. 8A). In particular, statistical analyses indicated that the effects of *IpLEA* for salt/osmotic stress tolerance on seed germination rates were remarkable, since the differences of seed germination rate and root length between *IpLEA OX* lines and WT showed significant (Fig. 8B,C). Additionally, to test the salt and osmotic tolerance of the plant seedlings, 4-day-old seedlings of the WT and *IpLEA OX* lines were transferred to MS medium containing different concentrations of NaCl (100, 150, and 200 mM) and mannitol (200, 300, and 400 mM), following which the root length was measured after cultivation under these treatment conditions for 7 d. When growing on control MS plates, no significant differences in root length appeared both in the WT and in transgenic lines plants (Fig. 9A). The root lengths of all of the transgenic plants were significantly longer than that of the WT plants when grown on MS plates containing NaCl (100, 150, and 200 mM) (Fig. 9A,B) or mannitol (200, 300, and 400 mM) (Fig. 9A,C), suggesting that the overexpression of *IpLEA* enhances salt/osmotic tolerance, thus improving seedling growth (Fig. 9).

**Figure 10:**
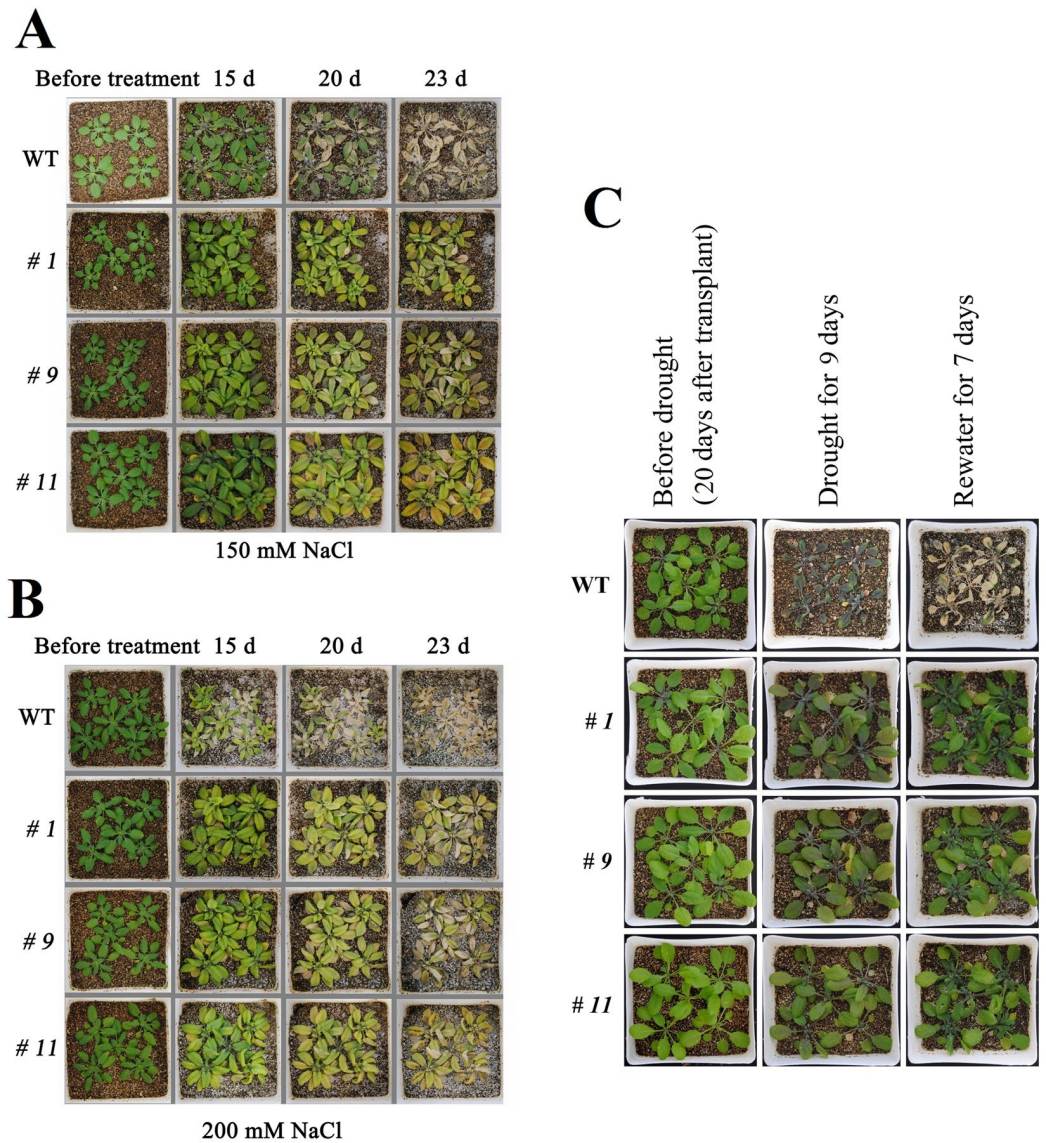
Photographs of *IpLEA* transgenic *OX* lines (*# 1*, *# 9*, and *# 11*) and WT plants grown in pots under normal and salt/drought conditions. (**A**) The effects of 150 mM NaCl on transgenic lines and WT; (**B**) The effects of 200 mM NaCl on transgenic lines and WT; (**C**) The effects of withholding water on the transgenic lines and WT.

Adult plants of the WT and *IpLEA OX* lines were also used to investigate the effect of the overexpression of *IpLEA* in Arabidopsis. Seven-day-old Arabidopsis seedlings were transplanted into well-watered vermiculite with nutrient solution from MS medium plates. Until 30 days of age (water was withdrawn for 20 d to gradually reduce the water content of the vermiculite), the WT and *IpLEA OX* line seedlings appeared to be growing well and in similar condition. Following this, the salt/drought tolerance assay began. We selected 150 and 200 mM NaCl treatments for the salinity stress experiment. Under normal conditions without salt challenges, no growth differences both in the transgenic lines (*# 1*, *# 9*, and *# 11*) and in the WT were observed. After the induction of salinity stress, almost all of the WT plants showed severe reduction in growth, and even death after 23 days’ high salinity stress, while the three transgenic lines remained alive (Fig. 10A,B). Furthermore, for the drought resistance assay, after an additional 9 d culturing without irrigation, severe dehydration of the leaves and wilting of the entire plant were observed, as well as the accumulation of anthocyanins in the leaves. The WT plants showed retarded growth, and even death, while the transgenic lines continued to grow slowly. After moderate watering for another 7 d, the transgenic lines recovered, while some WT plants exhibited a death phenotype (Fig. 10C). These results suggest that IpLEA may enhance the salt and drought stress resistance of transgenic Arabidopsis.

### Overexpression of *IpLEA* in Arabidopsis enhances oxidative stress tolerance

**Figure 11:**
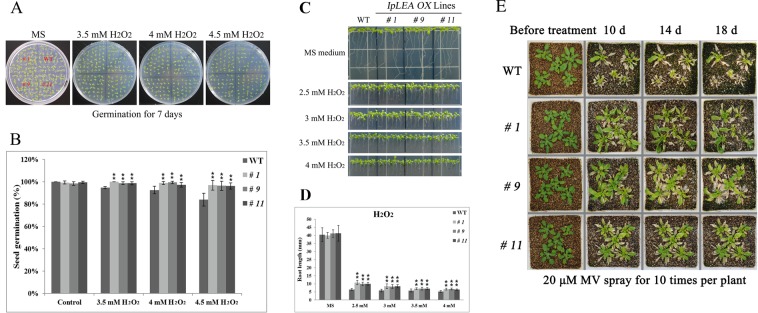
Oxidative stress analyses of *IpLEA* transgenic *OX* lines (*# 1*, *# 9*, and *# 11*) and WT with respect to seed germination rate, seedling root length, and adult plants. (**A**) Photographs of three transgenic lines and WT seeds germinated on MS medium or MS medium with H_2_O_2_ (3.5 mM, 4 mM, and 4.5 mM) for 7 d; (**B**) statistics for seed germination rates of the WT and *IpLEA* transgenic *OX* lines under H_2_O_2_ (3.5 mM, 4 mM, and 4.5 mM) for 7 d; (**C**) photographs of *IpLEA* transgenic *OX* lines and WT seedlings on MS medium or MS medium with H_2_O_2_ (2.5 mM, 3 mM, 3.5 mM, and 4 mM) for 7 d; (**D**) statistics for seedling root length of the WT and *IpLEA* transgenic *OX* lines under H_2_O_2_ (2.5 mM, 3 mM, 3.5 mM, and 4 mM) for 7 d; (**E**) oxidative stress analyses of the WT and *IpLEA* transgenic *OX* lines plants. Phenotypes of 3-week-old *IpLEA OXs* and WT plants that were treated with 20 μM MV for 18 d. Error bars indicate the ± SD based on three replicates. Asterisks indicate significant differences from the WT (control, Student’s *t* test, *P < 0.05 and **P < 0.01).

Since IpLEA showed obvious anti-oxidative capability both in yeast^26^ and in *E*. *coli* (Fig. 3B,C), we thus also assessed the possible anti-oxidative characteristics of IpLEA in plants. In brief, the seed germination rates of the WT and *IpLEA OXs* under H_2_O_2_ challenge were assessed. From Fig. 11A,B we can see that, under different H_2_O_2_ treatment concentrations (3.5 mM, 4 mM, and 4.5 mM), the three *IpLEA OXs* always exhibited a better seed germination status than the WT (Fig. 11A), and the seed germination rates were obviously higher in *IpLEA OXs* than in the WT (Fig. 11B). The root lengths of the three *IpLEA OXs* and WT were also measured under H_2_O_2_ challenge (2.5 mM, 3 mM, 3.5 mM, and 4 mM), and our results indicated that the overexpression of IpLEA in Arabidopsis improved the root growth of the seedlings under the oxidative stress caused by exogenous H_2_O_2_ (Fig. 11C,D).

To further confirm the role of *IpLEA* in regulating antioxidant mechanisms in adult Arabidopsis, the relationship between *IpLEA* and photo-oxidative stress was investigated by supplying MV to the *IpLEA OXs* and WT seedlings. MV is an herbicide that can generate highly reactive, oxygen-centered free radicals within the chloroplasts when plants are exposed to sunlight^32^. As shown in Fig. 11E, when the plants were supplied with MV, after 10 d, very little difference was observed between the WT and *IpLEA OXs*. In contrast, till 14 d and 18 d, the WT leaves were partly bleached under the 20 μM MV challenge and showed more withered than *IpLEA OXs* plants, and some of WT plants appeared lethal phenotype. Meanwhile, the transgenic lines remained alive and the central bud and young leaves were still green under the same concentrations (Fig. 11E). Our results indicated that the *IpLEA* transgenic lines demonstrated superior growth to the WT seedlings under oxidative stress.

### Overexpression of *IpLEA* affects ROS accumulation status under salt/drought stresses in Arabidopsis

**Figure 12:**
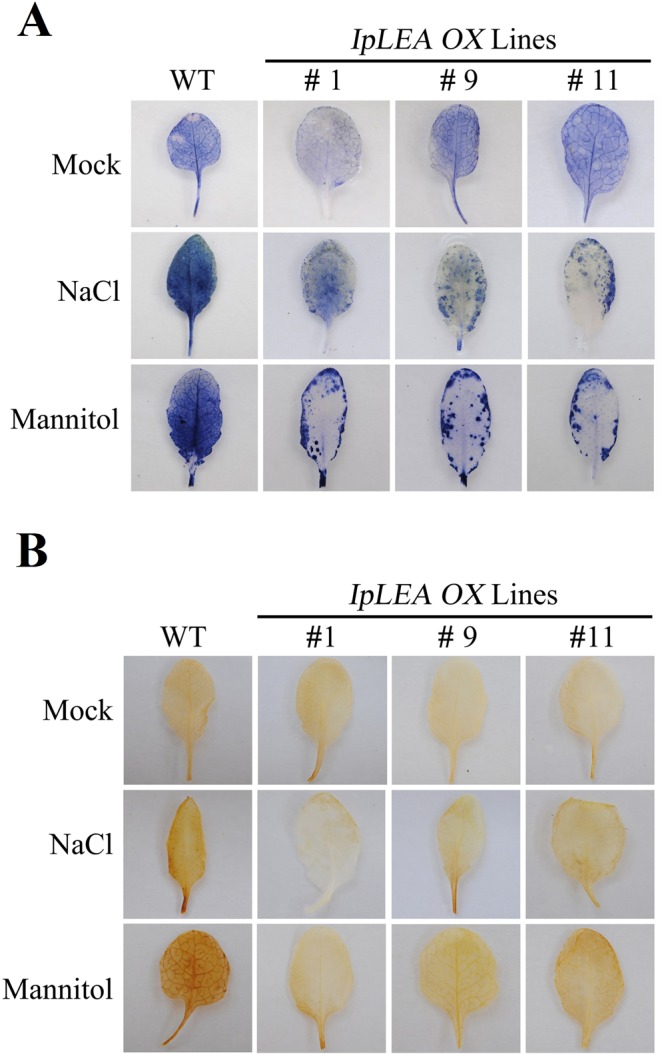
Oxidative stress analyses of the *IpLEA* transgenic *OX* lines (*# 1*, *# 9*, and *# 11*) and WT plants. Histochemical staining assays were used to detect H_2_O_2_ and O_2_^−^ in the leaves by NBT (**A**) or DAB (**B**) staining, respectively.

The accumulation of H_2_O_2_ and O_2_^−^ was also determined by DAB and NBT staining respectively, to further evaluate the ROS scavenging activity of the *IpLEA OX* lines, considering the previous oxidation resistance test for IpLEA in microorganisms (Fig. 3). As shown in Fig. 12, we measured the levels of ROS in the *IpLEA* *OX* lines and WT with or without salt (200 mM NaCl) or osmotic stress (300 mM mannitol). Compared to the WT, these three *IpLEA* *OX* lines showed significantly lighter NBT staining and less O_2_^−^ accumulation under salt and osmotic stress conditions (Fig. 12A). Similarly, DAB staining and quantification of H_2_O_2_ content revealed lower quantities of H_2_O_2_ in the *OX* lines than in the WT following NaCl treatment (Fig. 12B). These results showed that the *IpLEA* transgenic line plants scavenged more ROS than the WT plants, and that *IpLEA* further relieved the damage caused by the abiotic stresses, thereby increasing the resistance of the *IpLEA* transgenic plants.

### Overexpression of *IpLEA* affects the expression of stress-responsive genes

**Figure 13:**
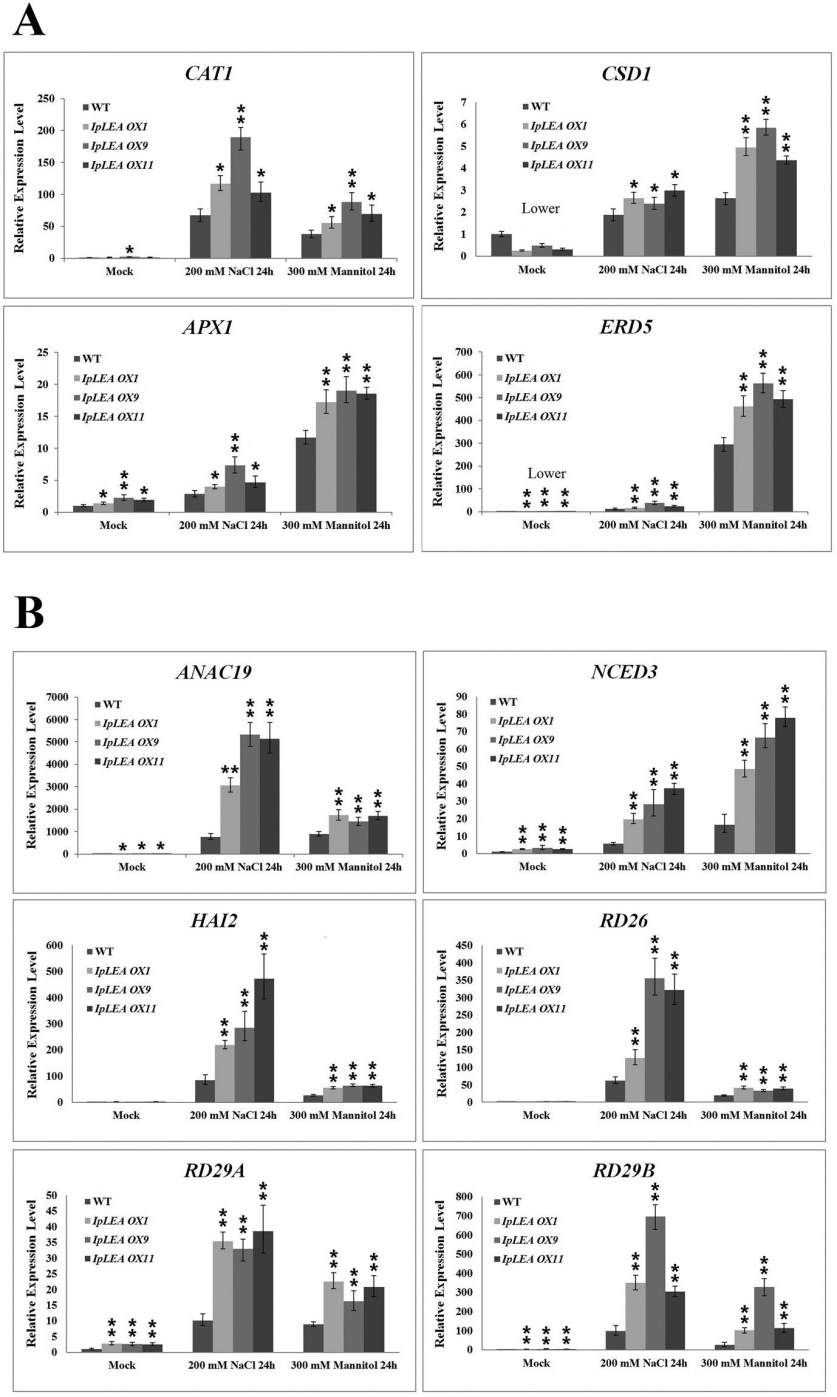
Analysis of the expression levels of ROS-related and stress-responsive genes in the *IpLEA* transgenic *OX* lines (*# 1*, *# 9*, and *# 11*) and WT plants by qRT-PCR under normal and salt/osmotic conditions. (**A**) *CAT1*, *CSD1*, *APX1*, and *ERD5*; (**B**) *ANAC19*, *NCED3*, *HAI2*, *RD26*, *RD29A*, and *RD29B*. Error bars indicate the ± SD based on three replicates. Asterisks indicate significant differences from the WT (control, Student’s *t* test, *P < 0.05 and **P < 0.01).

ROS staining analysis of the *IpLEA* *OX* plants suggested that the IpLEA protein conferred higher stress tolerance due to the stronger capacity of the transgenic plants then WT plants to eliminate the harmful ROS (Fig. 12) generated by abiotic stress. Here we also assessed the resistance of the *IpLEA* transgenic plants via the expression analysis of some antioxidant genes and stress marker genes. The qRT-PCR analysis revealed that the expression levels of some antioxidant-enzyme genes (*CAT1*, *CSD1*, and *APX1*) and a Pro biosynthesis gene (*ERD5*) were dramatically upregulated by salt/osmotic stress challenges (Fig. 13A). Additionally, some stress-responsive genes were up-regulated by water deficit and osmotic stresses, and were thus determined to be involved in regulating gene expression and hormone signaling, and activating protective responses in response to adversity in plants. The qRT-PCR analysis results of six stress-marker genes (*ANAC19*, *NCED3*, *HAI2*, *RD26*, *RD29A*, and *RD29B*) indicated that the expression of these genes in the transgenic plants was significantly higher than in the WT under oxidative stress (Fig. 13B).

## Discussion

As a seashore plant, *I*. *pes-caprae* is a halophyte that exhibits fairly good tolerance to seawater and aridity^24^. *I*. *pes-caprae* typically occurs in arid habitats with a lot of lime sands, as well as coastal beaches that are frequently submerged by seawater. Abiotic stresses, such as high salinity or dehydration, strongly influence the growth and development of *I*. *pes-caprae*, and thus this plant is considered to be a good germplasm resource for selective breeding due to its strong capacity to thrive under extremely saline and arid conditions.

In the present study, we isolated the *IpLEA* gene encoding a LEA_2 protein (containing PF03168 motif) from *I*. *pes-caprae* and verified its function. *LEA* genes have been shown to play pivotal roles in the abiotic stress responses of many plant species^2,8^. Although IpLEA showed some degree of hydrophilicity and IDP (intrinsically disordered protein) characteristics^8^, the GRAVY of IpLEA is relatively low (–0.373), which indicated that the hydrophilicity of IpLEA is not as high as other IpLEAs (IpDHN, –1.364)^27,28^. Accordingly, the 3D prediction by PHYRE^2^ (http://www.sbg.bio.ic.ac.uk/phyre2/html/page.cgi?id=index) also indicated that IpLEA contains several α-helixes and β-sheets (Fig. S2), and the instability index (II) of IpLEA is relatively low (29.09) than some typical IDPs (for example, the II values of IpDHN and IpASR are 67.66 and 36.82 respectively)^27,28^. It has been reported that LEA_2 subfamily proteins are atypical, since they are predicted to maintain a stable structure in solution^31^ and have a higher content of hydrophobic residues than typical LEAs^23^. In general, *LEA_2* genes in various plant species are believed to be associated with several abiotic stresses, such as salt or drought stresses^22,23,33^, and the heterologous over-expression of *LEA_2s* can increase stress tolerance in transgenic plants, which strongly suggests a role for LEA_2 proteins in stress tolerance^34^.

To gain insight into whether *IpLEA* is involved in the response to abiotic stresses or in regulating growth and development in *I*. *pes-caprae*, a series of expression pattern analyses were performed with untreated or treated *I*. *pes-caprae* tissues. As shown in Fig. 5A, *IpLEA* was expressed in all of the tissues in the *I*. *pes-caprae* plants. *IpLEA* mRNA was relatively highly accumulated in the roots of *I*. *pes-caprae* seedlings and adults, which indicated that *IpLEA* might be more involved in the response to external stress signals, since the roots are the organs that directly perceive high salinity or other osmotic stresses^35^. The *IpLEA* also presented high transcriptional level in mature leaves, petals, and developing seeds, which further showed that *IpLEA* might be involved in responding to water-deficit of these organs’ development or perceiving drought signals (Fig. 5A). Furthermore, *IpLEA* mRNA expression was strongly induced by multiple abiotic stresses, including salinity, dehydration, oxidative stress, cold treatment, and the dehydration-related hormone ABA (Fig. 5B–F). These data, therefore, suggest that the function of *IpLEA* is associated with water-deficit tolerance, probably by holding water molecular or ROS, and then reducing the damage by its protective roles to maintain the cellular basal metabolic activities. This conclusion is further supported by our previous results whereby the induced expression of the IpLEA protein in yeast could elevate the survival capability of yeast cells under salt and H_2_O_2_ stress^26^.

Given the specificity and uniqueness of the biological functions of LEA proteins, we propose that in some extreme halophytes, anhydrobiotic plants, or hardy plants, *LEA* genes might confer more specific or significant biological functions than in orthodox plants, or that the specific promoters show more salient stress-regulated features than in orthodox plants. In recent years, increasing reports on the gene functions, expression mechanisms, and global integration patterns associated with the adaptation of plants to their environment have tended to focus on wild plants, especially plants in extreme environments^36**–**38^. An earlier report on halophytes indicated that in addition to the functional genes, some inducible or constitutive promoters could be partially responsible for abiotic stress tolerance in plants^39^. As indicated in Fig. 1, the sequence of IpLEA only showed two amino acid differences from InLEA from the glycophyte *Ipomoea nil*. We proposed that the expressional regulation pattern of *IpLEA* mediated by the promoter might be an issue as crucial as the LEA protein *per se*. In the present study, the cloned *IpLEA* promoter region (1,495 bp) contained several potential *cis*-acting elements that respond to environmental stress, such as ABRE, MBS, and TC-rich repeats (Fig. 2). Furthermore, the expression of *IpLEA* in *I*. *pes-caprae* was also regulated by abiotic stresses and ABA (Fig. 5D). The promoter region contains several *cis*-acting elements that can bind to some specific stress-responding TFs, should be important implications to understand the transcriptional regulatory mechanism and gene expression pattern. Here, we can speculate that this putative *IpLEA* promoter could be a stress-responsive promoter, and the identification of *cis*-acting elements in the *IpLEA* promoter may help to provide insight into the molecular mechanism of the function of *IpLEA* and its further application in plant genetic engineering.

Numerous studies have focused on the regulatory mechanisms of the promoter for specific functional genes, rather than simply the protein sequence^40^. Due to the sessile nature of plants, under environmental change, plants must develop gene regulatory networks in order to adapt to the external environment, which can even influence their evolutionary trajectory. The promoter is pivotal for understanding the function of a gene, from which the associated mechanisms or some of the regulatory gene networks can be deduced^39^. The drought resistant plant *Populus euphratica* can survive in extremely arid environments, and the identified *PeNAC1* promoter showed obvious stress-inducible characteristics^41^. Two promoters of salt tolerant genes from the extreme halophyte *Salicornia brachiata*, *SbGSTU* and *SbNHX1*, have been suggested to play pivotal roles in the salt stress response^42,43^. The promoter of an atypical *LEA*, *SiLEA14* from foxtail millet (*S*. *italica*), also showed high salt/osmotic and ABA inducible features, which provides further evidence that the detailed salt/drought tolerant function of *SiLEA14*^23^. Based on our combined results regarding the promoter region of *IpLEA*, we can presume that the ABRE *cis*-regulatory motif binds ABA signal transcription factors and participates in the transcriptional regulation of *IpLEA* in response to ABA and osmotic stress signal transduction, as it does in other plants^44,45^. The MBS sites can bind MYB TFs and are suggested responding to drought stress and ABA signaling^46^, while TC-rich repeats *cis*-acting elements are involved in defense and stress responsiveness^47^. Our results provide insight into the molecular mechanism of the function of *IpLEA* in the extreme halophyte *I*. *pes-caprae*.

*E*. *coli* is an excellent experimental system for protein functional verification and has long been used as a powerful tool for exploring genetic function in multicellular organisms, especially for plant genes associated with osmotic stress^22,48^ or cold tolerance^49^. In the present study, after the induction of His-tag IpLEA protein in *E*. *coli*, we assessed the influence of IpLEA enhancing the stress tolerance of the transgenic *E*.*coli* strain *Rossetta* (DE3). Our research demonstrated that the His-tag IpLEA protein was rapidly and greatly induced in *E*. *coli* (Fig. 3A). Furthermore, our spot assay, CFU counting assay and bacteria growth curves of *E*. *coli* all indicated that IpLEA could distinctly improve salt/drought and antioxidative tolerance in the *E*. *coli* cells (Fig. 3B–D).

Since the *LEA* gene was first isolated from cotton seeds^5^, there has been increasing evidence that *LEAs* are extensively involved in physiological processes associated with abiotic stress, and particularly water deficit stress^1^. In general, the accumulation of LEA proteins or mRNA in plant cells has been associated with cellular desiccation process, including seed or pollen late development, anhydrobiotic plants, as well as abiotic stress tolerance, such as drought, low temperature, and high salinity in orthodox plant vegetative organs^2,4^. Numerous studies have shown that overexpressing *LEA* genes can improve the ability of plants to withstand diverse abiotic stresses^4^. To date, only a few reports exist on the functional verifications of the atypical *LEA_2*s involved in stress tolerance in plants, such as *SiLEA14* from foxtail millet^23^, *AtLEA14* from Arabidopsis^33^, *IbLEA14* from sweet potato^50^, and *SmLEA1* and *SmLEA2* from *S*. *miltiorrhiza*^22,51^. Here, we provide new functional evidence that a plant *LEA_2* member is involved in the adversity response and plays pivotal roles in increasing stress resistance, especially in the extreme halophyte *I*. *pes-caprae*.

Due to the diverse roles of LEA proteins in the response to abiotic stress, the cellular functional mechanisms of plant LEAs have been generalized into three patterns, namely, sequestering by binding H_2_O, ROS, ions, or sugars (I), binding membranes to maintain structural integrity (II), and safeguarding active enzymes by molecular shielding, as well as refolding misfolded proteins by acting as a disordered chaperone (III)^8^. In the present report, we focused on the anti-oxidative capacity of IpLEA and related it to the elevated tolerance to H_2_O_2_ in yeast^26^ and *E*. *coli* cells (Fig. 3). Transgenic Arabidopsis plants overexpressing *IpLEA* were generated in order to test the contribution of IpLEA to salt and drought tolerance (Figs. 8–10). The presence of either NaCl or mannitol in the MS medium can reduce water uptake by Arabidopsis, and accompanied by osmotic stress and ion toxic, leading to slower seed germination rates or root growth. When exposed to either of those components, our overexpressing plants demonstrated higher seed germination rates, faster root elongation, and improved growth compared with the WT Arabidopsis (Figs. 8 and 9), thereby indicating that stress tolerance was improved in the *IpLEA* overexpression transgenic plants. In addition, the *IpLEA* transgenic lines showed stronger ability to resist oxidative stresses than WT in seeds germination, seedling growth, and adult plants (Fig. 11). Changes of H_2_O_2_/O_2_^−^ distribution (Fig. 12) in the stress response in plants are also important markers for evaluating tolerance as they provide an indication of anti-oxidative capability. Furthermore, the regulation of ROS production and scavenging by the antioxidant defense machinery is an important indication of plant resistance^52–54^. Our results demonstrated that IpLEA could effectively prevent or minimize cellular oxidative damage, thus enhancing the salinity and drought tolerance of the plant. We also evaluated the gene expression of some anti-oxidative genes and ABA/stress-related genes, and the results indicated that the overexpression of *IpLEA* was responsible for the higher expression of these genes and subsequent stress tolerance (Fig. 13). Here, we proposed that IpLEA might act as a protective chaperone for specific TFs to activate the expression of resistance genes in plants.

In conclusion, we identified a novel atypical LEA_2 subfamily gene, *IpLEA*, from *I*. *pes-caprae*. Our current data, together with available over-expression assays in microorganisms and Arabidopsis, support a functional role for *IpLEA* in salt and drought stress tolerance. Our work shows that *IpLEA* exhibits inducible expression patterns in response to high salt, osmotic stress, ABA, cold, and oxidative stress, which indicates that the promoter of *IpLEA* is abiotic stress-responsive. IpLEA is localized in the cytosol, and the accumulation of IpLEA in cells elevated ROS scavenging capability and improved the transcriptional level of stress-related genes. Our findings suggest that this protein has a protective function in water-deficit and ROS-detoxification due to its water-holding capacity and ROS scavenging capacity, thereby affecting the expression of genes involved in salt/drought stress. Based on the results in this study, we speculated that the pleiotropic effects caused by the cellular accumulation of IpLEA could lead to enhanced cellular water balance and improved ROS homeostasis, resulting in improved cell viability and plant growth under drought and salinity stress. The results indicate that this IpLEA protein has a significant role in improving the tolerance and survival ability of plants or organisms under abiotic stress and could constitute a promising candidate for salt/drought tolerance transgenic plant research.

## Supplementary information

Supplementary Information
