# Supplementary material for: Isolation and characterization of an atypical LEA gene (IpLEA) from Ipomoea pes-caprae conferring salt/drought and oxidative stress tolerance
Source: Sci Rep. 2019 Oct 16;9:14838. doi: 10.1038/s41598-019-50813-w (PMC6796003; doi:10.1038/s41598-019-50813-w)

Supplementary information for

Isolation and characterization of an atypical *LEA* gene (*IpLEA*) from *Ipomoea pes-caprae* conferring salt/drought and oxidative stress tolerance

Jiexuan Zheng [1, 2], Huaxiang Su [1, 2], Ruoyi Lin [1, 3], Hui Zhang [1, 2], Kuaifei Xia [1], Shuguang Jian [1] & Mei Zhang [1]

[1]Key Laboratory of South China Agricultural Plant Molecular Analysis and Genetic Improvement & Guangdong Provincial Key Laboratory of Applied Botany, South China Botanical Garden, Chinese Academy of Sciences, Guangzhou, China; Center of Economic Botany, Core Botanical Gardens, Chinese Academy of Sciences, Guangzhou 510650, P.R. China. [2]College of Life Sciences, University of the Chinese Academy of Sciences, Beijing 100039, P.R. China. [3]College of Resources and Environment, University of the Chinese Academy of Sciences, Beijing 100039, P.R. China. Correspondence and requests for materials should be addressed to M.Z. (email: zhangmei@scbg.ac.cn).

Supplementary Fig. S1. The simple map of *IpLEA*-PRO/pBI101.2 (A) and *IpLEA*/pBIm (B).

Supplementary Fig. S2. The 3D structural diagram of IpLEA predicted by $PHYRE^2$ (http://www.sbg.bio.ic.ac.uk/phyre2/html/page.cgi?id=index).

Supplementary Fig. S3. The confirmation for *IpLEA's* over-expression in Arabidopsis plants. (A) DNA agarose gel electrophoresis for semi-quantitative RT-PCR and genomic PCR confirmation and (B) Quantitative RT-PCR test for *IpLEA OX* lines.

Supplementary Table S1. Primer sequences used in this study.

Supplementary Table S2. The physical and chemical properties of IpLEA protein.

Fig S1

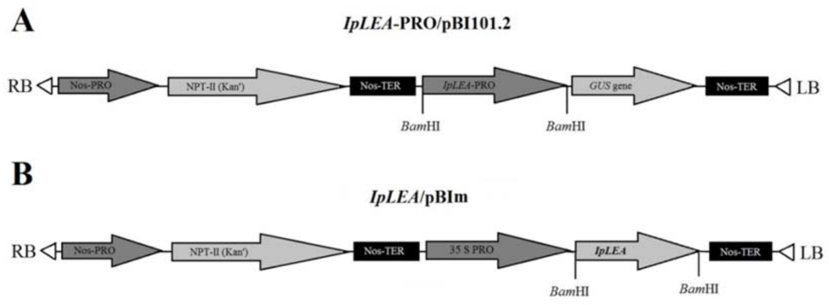

Fig S2

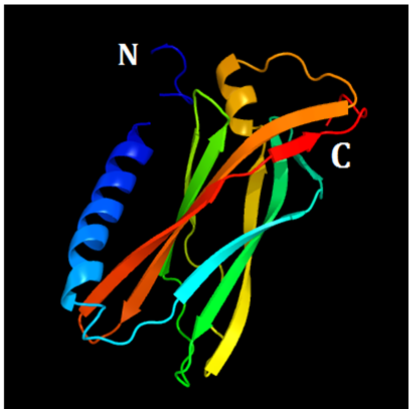

Fig S3

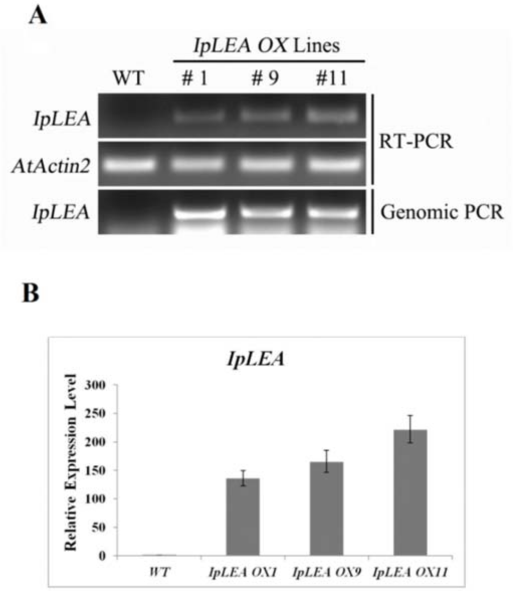

Tab. S1 Primer sequences used in this study.

Primer ID	Sequence (from 5′ to 3′)	Feature
IpLEAF	ATGGCATCGTCTGATAATCC	gene primer pair for sequence of *IpLEA*
IpLEAR	TCAATCCTCCTCATCATCAT	genomic DNA
IpLEASP1	GGGATCAGCCCCGAGATCAG	IpLEA gene-specific primers for genome
IpLEASP2	ACAGGATTTGGATTCTTCAC	walking to amplify promoter sequence of
IpLEASP3	AATCTCGGACACATCTGCAG	*IpLEA*
IpLEAPEF	AATGGGTCGCGGATCCATGGCATCGTCTGATAATCC	gene primer pair for construction of IpLEA-pET 28a, *Bam*HI site was underlined
IpLEAPER	GCTCGAATTCGGATCCTCAATCCTCCTCATCATCAT	
IpLEAGF	CTTGCTCCGTGGATCCATGGCATCGTCTGATAATCCA	gene primer pair for construction of IpLEA-pUC/EGFP, *Bam*HI site was
IpLEAGR	TGCTCACCATGGATCCATCCTCCTCATCATCATCTCC	underlined
IpLEARTF	ACCAACTGCAGATGTGTCCG	gene primer pair for qRT-PCR of *IpLEA*
IpLEARTR	CGTTCCAGCATCAGGGATCA	
IpUBQRTF	TCGACAATGTGAAGGCAAAG	gene primer pair for qRT-PCR of reference
IpUBQRTR	CTTGATCTTCTTCGGCTTGG	gene *IpUBQ*
CAT1RTF	CGCCATGCCGAAAAATACCC	gene primer pair for qRT-PCR of *CAT1*
CAT1RTR	CTTGCCTGTCTGAATCCCAGGAC	(At1g20630) in *Arabidopsis*
CSD1RTF	TGATGGAACTGCCACCTTCACA	gene primer pair for qRT-PCR of *CSD1*
CSD1RTR	ATGGCCTCCCTTTCCGAGGT	(At1g08830) in *Arabidopsis*
APX1RTF	GGACGATGCCACAAGGAT	gene primer pair for qRT-PCR of *APX1*
APX1RTR	CGACCAAAGGACGGAAAA	(AT1G07890) in *Arabidopsis*
ERD5RTF	GTCCTCTCTACCACACAAACTC	gene primer pair for qRT-PCR of *ERD5*
ERD5RTR	TGGACTCTTGGCATTTCCTAC	(AT3G30775) in *Arabidopsis*
ANAC19RTF	CAACTGTGGCTACCTGAAGACGG	gene primer pair for qRT-PCR of
ANAC19RTR	CAAACGAGTCAACACCATAACCCT	*ANAC19* (At1g52890) in *Arabidopsis*
NCED3RTF	GCTGCGGTTTCTGGGAGAT	gene primer pair for qRT-PCR of
NCED3RTR	TTGAGAAGACGATAATGGCGG	*NCED3* (At3g14440) in *Arabidopsis*
HAI2RTF	ACGGGCTATGGGACGTAGTG	gene primer pair for qRT-PCR of *HAI2*
HAI2RTR	ACACATGCGCACCATCGTA	(At1g07430*)* in *Arabidopsis*
RD26RTF	AGTTCGATCCTTGGGATTTG	gene primer pair for qRT-PCR of *RD26*
RD26RTR	ACCCGTTGCTTTCCAATAAC	(At4g27410) in *Arabidopsis*
RD29ARTF	GATATCGACAAGGATGTGCCG	gene primer pair for qRT-PCR of *RD29A*
RD29ARTR	GTATCCAGGTCTTCCCTTCGC	(At5g52310) in *Arabidopsis*
RD29BRTF	AAGGAGACGCAACAAGGG	gene primer pair for qRT-PCR of *RD29B*
RD29BRTR	ACGGTGGTGCCAAGTGAT	(At5g52300) in *Arabidopsis*
ACT2RTF	GGTAACATTGTGCTCAGTGGTGG	gene primer pair for qRT-PCR of
ACT2RTR	AACGACCTTAATCTTCATGCTGC	reference gene *AtActin2* (At3g18780)
IpLEAOXF	GGACTCTAGAGGATCCATGGCATCGTCTGATAATCCA	for cloning the full-length ORF of *IpLEA* and construction of IpLEA-pMD, *Bam*HI site was
IpLEAOXR	GTCGACCCGGGGATCCTCAATCCTC	underlined

	CTCATCATCAT	
IpLEAProF	CGACTCTAGAGGATCCAATCTCGGACACATCTGCAG	for cloning the full-length promoter and construction of IpLEA-Pro:GUS, *Bam*HI site was underlined
IpLEAProR	ACCTACCCGGGGATCCACCTTCTCACAAGCTGAGAT	

Tab. S2 The physical and chemical properties of IpLEA protein.

**Physical and chemical properties**	**IpLEA**
No. of amino acids	313
Molecular weight (kD)	34.92
Theoretical pI	4.78
Total no. of negatively charged residues (D+E)	61
Total no. of positively charged residues (R+K)	39
Grand average of hydropathicity(GRAVY)	–0.373
instability index (II)	29.09

Figure 3A The full-length gels/blots of this manuscript.

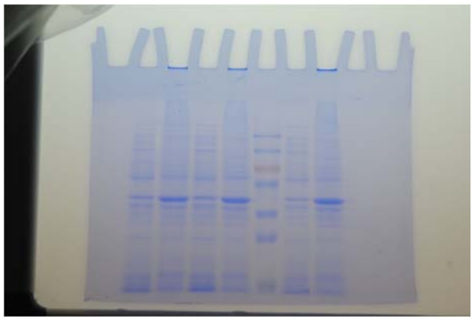

Fig S3 The full-length gels/blots of this manuscript.

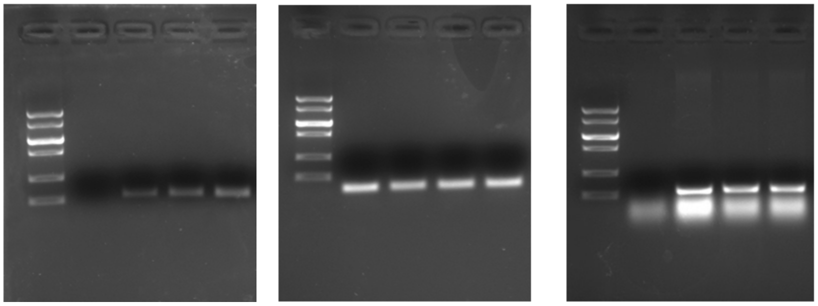

Supplement: Supplementary file 1 — Supplementary Information [file 41598_2019_50813_MOESM1_ESM.pdf]
